# Delay of Germination-1 (DOG1): A Key to Understanding Seed Dormancy

**DOI:** 10.3390/plants9040480

**Published:** 2020-04-09

**Authors:** Néstor Carrillo-Barral, María del Carmen Rodríguez-Gacio, Angel Jesús Matilla

**Affiliations:** 1 Departamento de Biología, Facultad de Ciencias, Universidad de A Coruña, Campus Zapateira, 15071-A Coruña, Spain; n.carrillo@udc.es; 2 Departamento de Biología Funcional (Área Fisiología Vegetal), Facultad de Farmacia, Universidad de Santiago de Compostela, 15782 Santiago de Compostela, Spain; mdelcarmen.rodriguez.gacio@usc.es

**Keywords:** DOG1, seed dormancy, ABA, ethylene, clade-A PP2C phosphatase (AHG1, AHG3), after-ripening, asDOG1, heme-group

## Abstract

DELAY OF GERMINATION-1 (DOG1), is a master regulator of primary dormancy (PD) that acts in concert with ABA to delay germination. The ABA and DOG1 signaling pathways converge since DOG1 requires protein phosphatase 2C (PP2C) to control PD. DOG1 enhances ABA signaling through its binding to PP2C ABA HYPERSENSITIVE GERMINATION (AHG1/AHG3). DOG1 suppresses the AHG1 action to enhance ABA sensitivity and impose PD. To carry out this suppression, the formation of DOG1-heme complex is essential. The binding of DOG1-AHG1 to DOG1-Heme is an independent processes but essential for DOG1 function. The quantity of active DOG1 in mature and viable seeds is correlated with the extent of PD. Thus, *dog1* mutant seeds, which have scarce endogenous ABA and high gibberellin (GAs) content, exhibit a non-dormancy phenotype. Despite being studied extensively in recent years, little is known about the molecular mechanism underlying the transcriptional regulation of *DOG1*. However, it is well-known that the physiological function of DOG1 is tightly regulated by a complex array of transformations that include alternative splicing, alternative polyadenylation, histone modifications, and a *cis*-acting antisense non-coding transcript (*asDOG1*). The DOG1 becomes modified (i.e., inactivated) during seed after-ripening (AR), and its levels in viable seeds do not correlate with germination potential. Interestingly, it was recently found that the transcription factor (TF) bZIP67 binds to the *DOG1* promoter. This is required to activate *DOG1* expression leading to enhanced seed dormancy. On the other hand, seed development under low-temperature conditions triggers *DOG1* expression by increasing the expression and abundance of bZIP67. Together, current data indicate that DOG1 function is not strictly limited to PD process, but that it is also required for other facets of seed maturation, in part by also interfering with the ethylene signaling components. Otherwise, since DOG1 also affects other processes such us flowering and drought tolerance, the approaches to understanding its mechanism of action and control are, at this time, still inconclusive.

## 1. Introduction

The seed is a key entity in the life cycle of higher plants. To this end, it enables and ensures its survival by acquiring the primary dormancy (PD) during the maturation phase. PD is defined as the incapacity of a viable seed to complete germination despite the conditions are favorable [1]. Dormancy is hormonally induced, maintained, and strictly regulated by the modulation of suitable hormonal signaling networks [1]. Seeds can detect spatial and temporal environmental conditions (e.g., temperature, O_2_ and light) [1,2,3,4]. PD dormancy is a notable agronomic feature. Thus, low levels may produce pre-harvest sprouting, while high levels inhibit the rapid and uniform germination rate. In both cases, there is a reduction of crop production [1,4,5]. PD is established and maintained in the viable dry seed, and throughout several molecular paths (e.g., after-ripening (AR) and exposure to gentle cooling) and can be broken gradually [6,7]. On the other hand, non-dormant seeds of various species have been reported to achieve secondary dormancy (SD) upon exposure to unfavorable conditions for germination [1]. SD occurs essentially after seed dispersal and may be induced by environmental interactions or other special conditions.

*DELAY OF GERMINATION1* (*DOG1*), a heme-binding protein, was identified in seeds of *A. thaliana* by using a biparental recombinant inbred population derived from a cross between the deeply dormant ecotype Cape Verde Islands (Cvi-0; high *DOG1* expression) and a weakly dormant ecotype Landsberg *erecta* (Ler-0; low *DOG1* expression). By analyzing this recombinant inbred line (RIL) population, seven dormancy related quantitative trait loci (QTLs) were identified [8,9]. The first of these QTLs cloned was *DOG1* which turned out to have a great impact on PD level (i.e., became the most important regulator of PD in Arabidopsis) [2,9]. *DOG1* is mainly expressed in seeds [10]. Despite being studied extensively over the last fifteen years [10], the precise molecular mechanism underlying the regulation of DOG1 still has gaps [10,11,12]. Interestingly, Li et al. (2019) provides mechanistic insights into how ethylene (ET) signaling controls PD via DOG1 regulation. Detailed comments on this striking work are included below.

**Figure 1:**
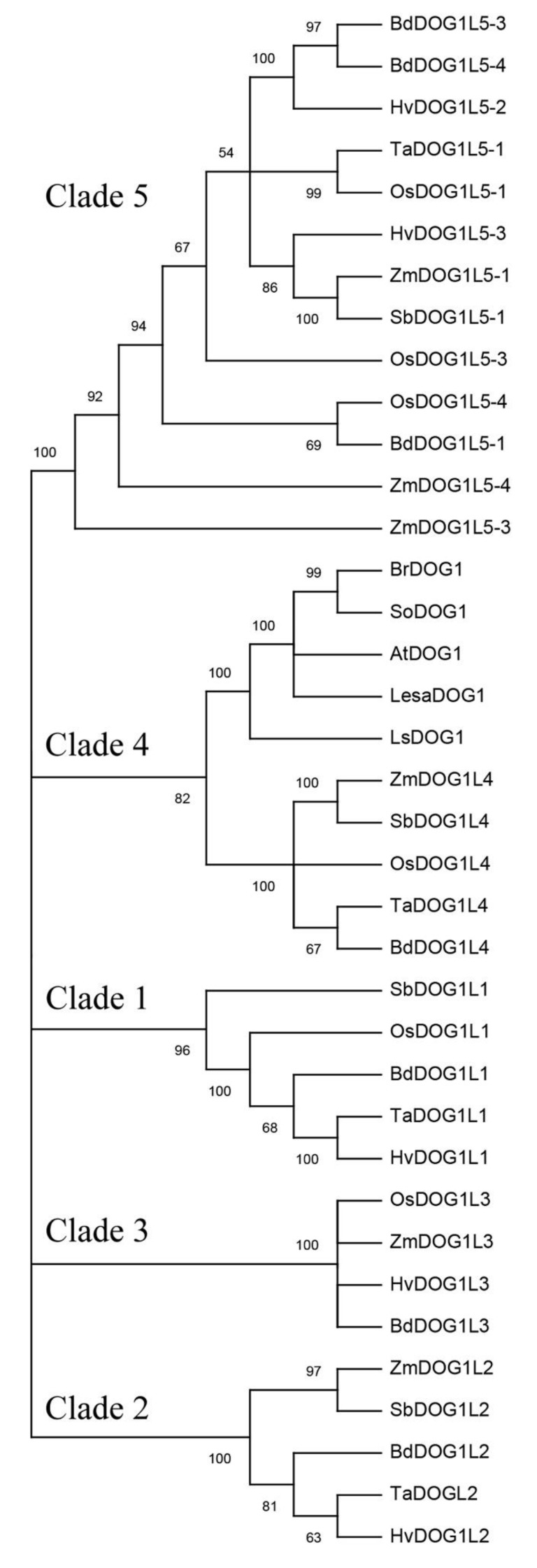
Phylogenetic tree of DOG1. Deduced DOG1 and DOG1-like amino acid sequences of *Arabidopsis thaliana* (AtDOG1, AtDOGL1-5); deduced DOG1 amino acid sequences of *Brassica rapa* (BrDOG1), *Lactuca sativa* (LsDOG1), *Lepidum sativum* (LesaDOG1), *Sisymbrium officinale* (SoDOG1); and the homologs in cereals *Brachypodium distachyon* (BdDOG1L1 to L5), *Hordeum vulgare* (HvDOG1L1, L2, L3, and L5), *Oryza sativa* (OsDOG1L1, L3, L4, and L5), *Sorghum bicolor* (SbDOG1L1 to L5), *Triticum aestivum* (TaDOG1L1, L2, L4, and L5), and *Zea mays* (ZmDOG1L2 to L5) were included. Tree was constructed with the neighbor joining method (1000 bootstrap repetitions) using MEGA X software. Only branches with a value above 50% are shown. An additional table indicates the accession numbers of the sequences included in the tree (Figure S1 and Table S1).

*DOG1* is a member of a small gene family. There are five *DOG1-LIKE* (*DOGL*) genes—*DOGL1*, *DOGL2*, *DOGL3*, *DOGL4,* and *DOGL5*—in the Arabidopsis genome [1]. The first four *DOGL* genes are located on chromosome 4 next to each other, while *DOGL5* was found on chromosome 3. The DOG1 family in Arabidopsis has several conserved domains whose functions are still under study [1,5]. DOGL1, DOGL2, and DOGL3 are relatively like DOG1, whereas DOGL4 and DOGL5 show only 28 and 30% similarity with DOG1 in amino acid sequence, respectively [1]. In parallel to Arabidopsis, *DOG1* genes have been found in other species of Brassicaceae [7,11,12] and some of *Lactuca* [13]. The amino acid sequences similarity among the studied dicot DOG1 is high [7,14]. Other authors have described *DOGL* genes in many monocot species, such as *Hordeum vulgare*, *Triticum aestivum*, *Oryza sativum*, *Zea mays*, *Sorghum bicolor,* and *Brachypodium distachyon* [5,14,15] (Figure 1, Figure S1, Table S1). A high number of *AtDOG1* homologues have been described in cereals. DOG1L1-4 in cereals show some functional conservation since they induce dormancy when ectopically expressed in Arabidopsis. However, the amino acid sequences of cereal DOG1L do not have much similarity with those of dicot plants. Although genes with some degree of similarity in amino acid sequence to *AtDOG1* were known to be present in species other than Arabidopsis, because of their low similarity to *DOG1* it remained to be answered whether these genes actually functioned in the regulation of PD in a broad range of species, or only in Arabidopsis and its close relatives [11]. *AtDOG1* and its homologous genes would be good candidates to try to manipulate PD in the near future. It has been found that *DOG1* expression and PD are controlled by a *cis*-acting antisense transcript (*asDOG1*) [16]. As will be described later, *asDOG1* expression is strongly suppressed by both ABA and drought, resulting in the release of antisense-dependent silencing of *DOG1* [16]. Moreover, it was shown that DOG1 is also involved in the seed maturation programme, seed longevity, PD release, and germination timing, this last property being an important adaptive trait controlled by seed dormancy. At this time, the detailed involvement of DOG1 in all these cited programmes is not yet well understood [9,10,11,13]. However, a notable function of DOG1 may be the induction of PD including the temperature-dependent PD [10,17,18,19,20,21,22]. Thus, it is known that the lower the seed maturation temperature, the higher the degree of dormancy. Concomitantly, DOG1-mRNA and protein levels also increase [17]. That is, *A. thaliana* accessions located in colder climates tend to initiate DOG1 expression earlier during seed maturation. *DOG1* is involved in the induction of PD in response to cool seed-maturation temperature experienced by the mother plant. Therefore, DOG1 is likely to exhibit environmental sensitivity [19]. Since DOG1 also affects flowering and drought tolerance, DOG1’s study becomes much more convoluted [13,22,23,24].

This review focuses on the reasons why DOG1 is being considered as a key signaling molecule to coordinate the seed life and very specifically the acquisition and loss of its PD.

## 2. DOG1 and Its Vital Functions during the Seed Life Cycle

In the last years, numerous studies have proved that the functions of DOG1 in plants are well conserved. Despite its well-documented implication in PD, DOG1 molecular task still needs much more attention [5,14,25,26].

### 2.1. DOG1 in Seed Maturation and Dormancy

**Figure 2:**
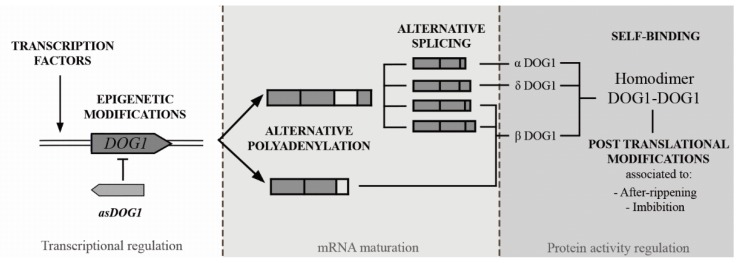
Different molecular mechanisms regulating the gene expression and protein activity of DOG1. The transcription of *DOG1* is regulated by epigenetic modifications and probably by TFs. Additionally, the transcription of a noncoding antisense sequence (asDOG1) acts as a negative regulator of *DOG1* expression. Two different precursor mRNAs are formed due to the existence of two polyadenylation sites in Arabidopsis. The precursor mRNAs are processed to five different mature mRNA by alternative splicing and lately translated to three different protein isoforms (three of the five mRNA encode the same protein isoform). DOG1 binds itself to form homodimers and can suffer post-translational modifications associated to AR and germination processes. However, the specific nature of these modifications is still unknown.

Seed maturation is a crucial phase of seed development that comprises several developmental processes such as reserve accumulation (e.g., seed storage proteins, SSPs), cessation of embryo growth, acquisition of desiccation tolerance, and PD [27,28]. Although there are many transcription factors (TFs) specifically associated with seed maturation, only ABI3, FUSCA3 (FUS3), LEAFY COTYLEDON 1 (LEC1), and LEC2 have been described as key regulators in Arabidopsis [1,4]. Mutations in these TFs produce alterations in seed maturation, affect accumulation of SSPs, and alter ABA sensibility and PD (e.g., *abi3-lec1* and *abi3-fus3* show an important reduction in SSPs content and present severe viviparism) [1,4,11]. The induction and release of PD depend on environmental conditions (e.g., temperature, light, and cold) and endogenous regulators (e.g., hormones, regulatory proteins, and chromatin status) [20,25,28]. It is noteworthy that the mechanism of endogenous plant hormonal regulation is suggested to be highly conserved in PD and germination processes. In reference to DOG1, its dimerization (Figure 2) is essential for its capability to impose PD [26]. However, it remains unclear how self-dimerization is involved in DOG1 function. Specifically, the expression of *DOG1* is absolutely required for the induction of PD. Conversely, DOG1 is reduced in fully mature dry seed [10,16,23] and *DOG1*-mRNA is nearly undetectable in seedlings [1,16]. It is well-known that during late seed maturation an accumulation of the raffinose family oligosaccharides (RFO), an increase of SSPs, heat-shock proteins (HSPs), and late embryogenesis abundant (LEA) proteins exist [28]. Transcriptomic and metabolomic analyses of *dog1-1* mutants revealed a decrease in HSP, LEA, and RFO. The expression of ABI5 is involved in this decrease [23,29,30]. It is striking that *dog1-1* mutant has reduced PD and reduced longevity. This fact suggests a positive correlation between both processes [9,31]. Several QTLs related to seed longevity have been identified [31]. The QTLs identified for longevity and PD do not necessarily co-locate, proposing that the natural variations of these two characteristics are regulated by different genetic mechanisms. The observation of a negative correlation between PD and longevity strongly suggests that seeds are capable of extending their life span either by PD or by an active longevity mechanism [26]. Furthermore, the analysis of *dog1-1 abi3-1* suggests that DOG1 may control the chlorophyll degradation and SSPs accumulation through an interaction with ABI3. These data also suggest that DOG1 and ABI3 work in parallel to stimulate maturation [23]. Moreover, individual analysis of *dog1* and *dog1 abi3* mutants suggests that DOG1 function is not limited to PD but that it is required for several aspects of seed maturation [23]. Likewise, variations in the *DOG1* expression during the PD seem to be partially due to an epigenetic regulation (i.e., histone methylation) (Figure 2), as will be discussed below.

ABA induces and maintains PD, while GAs releases PD and induces germination. Accordingly, the dormancy status is regulated by the balance ABA: GAs, which will be used for the seed to interpret the environmental conditions and therefore stay dormant or germinate [1,32,33,34,35]. Cold temperatures exposure of maternal plants during seed maturation induces an increase in both DOG1 and PD status [20]. Thus, accessions of Arabidopsis from the north of Europe are less dormant and exhibit lower levels of *DOG1* transcripts than the southern ones [17]. On the other hand, the level of dormancy cycling in the field is not quantitatively related to ABA. Accordingly, the analysis of a set of accessions of four distinct geographical areas revealed that *DOG1* contributes to local adaptation [36,37,38]. DOG1 is important to determine the deep dormancy phase and it could act as part of a thermal-sensing system to affect PD level by altering the ABA sensitivity [34]. A study with buried Arabidopsis seeds showed dynamic changes in SD intensity, which had a strong correlation with *DOG1* expression level [33,36]. Murphey et al. [19] proposed that *DOG1* is a relevant gene in the induction of SD in response to temperatures. In some seeds, DOG1 induces SD because of the warm and prolonged cold stratification in the course of seed imbibition [20,39]. Finch-Savage and Footitt indicated in their recent update on PD [34] that the thermo-inhibition of germination was DOG1 dependent and not reliant on an increased amount of ABA in lab conditions; while DOG1 is of decisive importance to dormancy cycling in field conditions in addition to its importance in determining the extent of PD. Besides, germination only occurs in the light if DOG1 expression is low as a result of chromatin remodeling and the level of active DOG1 protein is reduced [37].

### 2.2. DOG1 Takes Part in Key Molecular Mechanisms Regulating Seed Dormancy

Recent studies in *Lactuca sativa* and *A. thaliana* reported that DOG1 stimulates the temperature-dependent PD by affecting the levels of determined microRNAs [13]. Thus, DOG1 can regulate PD by means of an influence on generation and/or action and processing of miR156 and miR172 microRNAs [1,13]. miRNAs are endogenous small non-coding RNAs that act as post-transcriptional regulators of gene expression in animals and plants by targeting mRNAs for cleavage or translational repression. miR156 accumulates in high levels during seed development and it is progressively lost during seed storage as a result of RNA oxidation [40,41]. RNA oxidation can be carried out by reactive oxygen species (ROS) generated in dry seeds, particularly by hydroxyl radicals [41]. Seed alleviation of PD during AR is associated with mRNA oxidation which is prevented when seeds are maintained dormant [40]. Therefore, DOG1 may play a role in repositioning the miR156 levels during seed development [22], which would provide a timing mechanism for seed AR via loss of the inhibitory effect of stored miR156 on germination. In short, DOG1 functions as an important molecular integrator that exerts its effects on developmental phase changes, at least in part through miRNA-regulated pathways [22].

The overexpression of *AtMIR156* causes the stimulation of PD, but *AtMIR172* diminishes it [13]. Interestingly, the expression of genes involved in miR156 and miR172 processing was lowered in *A. thaliana* and *L. sativa* dry viable seeds from *dog1* mutants, whereas the overexpression of MIR172 reduced seed PD [13]. It is highlighted that the essential role played by miR156 and miR172 in sensing and integrating environmental changes, and the role of DOG1 in affecting the relationship miR156/miR172 thus play a significant role in seed development [13]. Besides, small RNA library of Arabidopsis seeds obtained from the field in mid-summer (low dormancy) and mid-winter (high dormancy) possessed abundant miR156 levels at both seasonal periods. These data suggest that DOG1 maintains high miR156 levels in soil seed bank over the spatial sensing phase till PD release [33,34]. These and other recent revised results seem to fall under the hypothesis that DOG1 is part of a thermal sensing mechanism in the regulation of DOG1 transcription [1,34]. That is, DOG1 transduces environmental effects during maturation to alter depth of dormancy thus linking DOG1 and dormancy cycling. However, since *DOG1* expression follows environmental cues, DOG1 does not appear to directly determine the pattern of dormancy cycling [34,36]. In other words, although a key role of *DOG1* was detected in determining the depth of dormancy, it was still not a direct role for *DOG1* in generating altered seasonal patterns of seedling emergence [36]. Therefore, it remains unclear whether variation in DOG1 expression can drive variation in dormancy cycling behavior.

**Figure 3:**
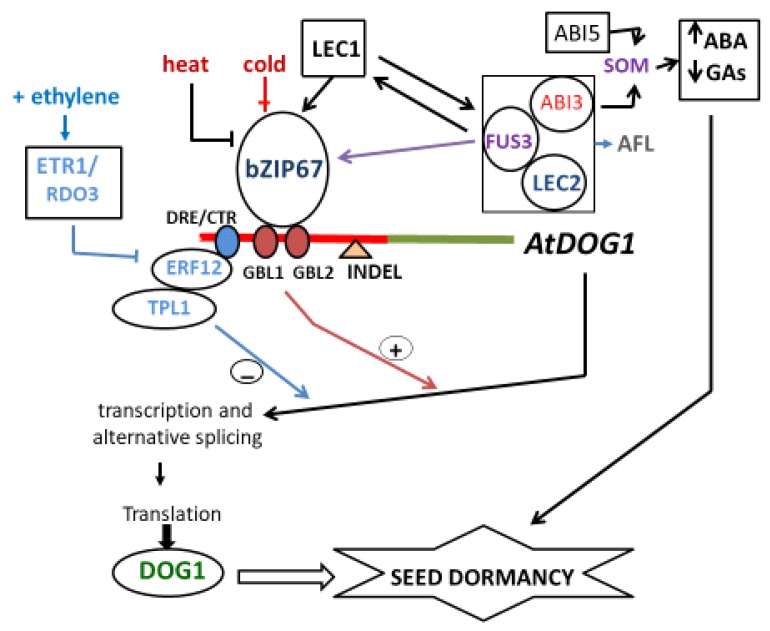
A model for the transcriptional regulation of *DOG1* gene by ethylene and low temperatures (promotor in red; coding region in green) to induce PD in *A. thaliana* (adapted from Li et al. 2019 [44]; Carbonero et al. 2017 [45], and Breeze et al. 2019 [46]). Downstream of the GBL1 *cis*-element is INDEL, a 285 bp sequence. INDEL is present in *DOG1* promoter of Ler-0 accession (weakly dormant). However, INDEL is absent in Cvi-0 ecotype (strong dormant). INDEL was found to directly affect the ability of bZIP67 to transactivate *DOG1* in vivo and may explain the observed variation in *DOG1* transcript levels, and consequently dormancy, between ecotypes. Such natural allelic variation in *DOG1* coupled with *DOG1* expression plasticity confers substantial adaptive significance in the field, where seasonal environmental factors can vary greatly, and the optimal timing of seed germination is primordial. ETR1: Ethylene Response-1; DRE/CRT: Dehydration-Responsive Element/C-Repeat; TPL1: Topless-1; ERF12: Ethylene Responsive TF-12; GBL1 and GBL2: G-Box-Like-1 and 2; SOM: Somnus.

DOG1 has been recently shown to physically interact with two phosphatases (ABA-HYPERSENSITIVE GERMINATION 1 and 3; AHG1 and AHG3) to functionally block their essential downstream roles in the release of seed PD [25,42] (Figure 3). AHG1 and AHG3 belong to the clade-A type 2C PP2C family (i.e., with nine members in Arabidopsis) and act as negative regulators of ABA signaling and seed PD [25,42]. As a result, seed PD and ABA sensitivity are increased not only when DOG1 protein levels are enhanced [10] but also in an *ahg1 ahg3* double mutant [25]. Likewise, *ahg1-1ahg3-1hai3-1* triple mutant has a deeper dormancy and also proposed that at least AHG1, AHG3, and HAI3 are involved in the regulation of PD [42]. In addition, DOG1 controls PD by suppressing the action of specific PP2C phosphatases, which function as a convergence point of the ABA and DOG1 pathways [25]. On the other hand, the binding of DOG1 to heme group is essential for DOG1 task during PD [42]. Heme is an iron-binding protoporphyrin IX that regulates diverse biological activities (e.g., signal transduction); but the study of its role in ABA signaling is still in very early stages. Binding of DOG1 to AHG1 and heme seem to be an independent processes, though both are essential for DOG1 function in vivo [40]. Likewise, the role of the redox state of the cell in the control of seed PD is an unexplored research field [43]. We will return to these last topics later.

### 2.3. Strict Regulation of DOG1 during Seed Dormancy

In *A. thaliana*, the *DOG1* gene is composed of 3 exons and 2 introns and is alternatively spliced of the second intron thus producing five transcript variants [26] and resulting in only three different proteins since translation of *β* and *γ* transcripts generate the same protein (Figure 2). The abundance of each *DOG1-mRNA* is different; but the proportion among them remains almost unaltered in different dormant and non-dormant accessions [2]. Recently, a short *DOG1-mRNA* was described [3]. This major form, named *ε*, excludes the third exon and incorporates a part of the second. The nascent mRNA-ε is formed by alternative polyadenylation and not by differential splicing [3,47]. The level of *DOG1* transcripts is strongly decreased throughout the final part of seed maturation, though the abundance of DOG1 protein does not diminish [10]. DOG1 α, β, γ, δ, and ε isoform proteins are functional [26]. DOG1-α, DOG1-β, and DOG1-δ were located in the nucleus and DOG1-β is much more abundant than the other isoforms [26]. Interestingly, the regulation of DOG1 protein accumulation by alternative splicing could be part of a mechanism to fine-tune seed dormancy (Figure 2). The DOG1 abundance might be explained by the existence of an altered ratio between *DOG1* transcripts. Thus, the abundance of the *DOG1-δ* at the final of maturation is higher compared to *DOG1*-β, which could increase the DOG1 protein although a general decrease in *DOG1* transcript levels is produced [26]. Interestingly, the solid results obtained by the Swiezewski’s group suggest that the accumulation of short isoform of DOG1 ε-protein is enough to generate a dormancy phenotype. That is, the short *DOG1-mRNA* is translated in vivo and the generated DOG1 protein is also functional in controlling seed PD establishment [3]. The short ε-mRNA is the most abundant in *A. thaliana*, resulting in the same protein as β and γ-mRNA. This short isoform of DOG1 protein is better conserved than the other two, as the third exon is highly variable or even lost in other species and is also the most active in PD induction. On the other hand, Nakabayashi et al. [26] described that all the DOG1 isoforms show self-binding properties, and the loss of this capacity does not alter protein levels but leads to non-dormant phenotypes. In other words, DOG1 protein function is enhanced by its self-binding property. Thus, formation of dimers among different DOG1 isoforms would be necessary for the proper regulation of DOG1 functioning. Interestingly, *DOGL3* and *DOGL5* (*DOGL5.2*, alternative spliced form like *DOG1*) also bind to PP2C, and overexpression of *DOGL3* causes ABA hypersensitivity in seed germination, like *DOG1*, whereas *DOGL5* overexpression does not result in increased ABA sensitivity in seeds [40]. These and other features lead us to conclude that, *DOG1* and *DOGL*s do not seem to share a great deal of redundancy in terms of PD imposition.

It was shown that natural variation for DOG1 binding efficiency provides variation in PD [10,16,26]. These natural variations in several Arabidopsis accessions may be part of a system to adjust the PD [26]. However, more research is still required to clarify the exact role of the different DOG1 protein isoforms in plants. Finally, and given that *DOG1* is extensively regulated [1,3,9,13,25,42,47], recent relevant evidence demonstrated that asDOG1, a long non-coding antisense RNA from DOG1 in Arabidopsis, suppresses *DOG1* expression during seed maturation in *cis* and promotes germination [16,23]. This *DOG1* antisense partner originates close to the *DOG1* proximal polyadenylation. The presence of a conserved region in the second intron of *AtDOG1* led to the discovery of a natural noncoding antisense RNA (asRNA) (Figure 2). This conserved site, unexpected in a non-coding region, contains a promoter that extends approximately from the ending of the second exon to the transcription start site of *AtDOG1*. The transcription of *asDOG1* is independent of the *DOG1* promoter, and as it has been described for other genes, the asRNA acts as a negative regulator of *DOG1* sense transcription and expression [16]. Although the *asRNA* has a 5′ cap and is polyadenylated and stable, it seems, as said before, to regulate *DOG1* expression in *cis*, the antisense transcription process being more important as a repressor than the *asRNA* transcript itself [16]. The fact that *asDOG1* originates from close to the transcription termination site of the sense gene, raises the question of how this proximity affects antisense promoter activity. Although *asDOG1* is present in seeds, especially in desiccation phase, it is more abundantly transcribed in seedlings, meristems, and young leaves. The addition of ABA downregulates the transcription of *asDOG1* in vegetative tissues and allows *DOG1* expression. Moreover, when the promoter of *asDOG1* is mutated, the addition of ABA is unable to induce *DOG1* expression. This ABA-dependent *asDOG1* transcription is particularly important for the role of DOG1 in the induction of drought tolerance. Yatusevich et al. (2017) [23], in a sophisticated work on epigenetic regulation of DOG1 in *A. thaliana*, showed that, (i) *asDOG1* suppresses *DOG1* expression in both seeds (i.e., prevents PD) and leaves (i.e., drought response) and that *dog1* mutants are more susceptible to drought; (ii) *asDOG1* expression is powerfully inhibited by ABA; (iii) the ability of the antisense promotor to respond to ABA is absolutely required for the regulation of *DOG1* expression by ABA; (iv) this group advances the possibility that DOG1 may regulate PD through similar mechanisms involving the ABA pathway; and (v) in the absence of *asDOG1*, *DOG1* is constitutively highly expressed in leaves [23]. Among other deductions, it is interesting to conclude that DOG1, in addition to participating in seed PD, also has other roles in plants. Finally, *asDOG1*-mediated control of *DOG1* expression and seed PD appear to be *cis*-restricted, suggesting a mechanism that may involve *asDOG1* transcription rather than arising RNA [16,23].

## 3. ABA and Ethylene Signaling during DOG1 Tasks

### 3.1. ABA and DOG1 Work Cooperatively

The critical role of ABA in the induction of PD is currently unquestionable [33]. ABA and DOG1 seem to act in concert to carry out the PD, a striking event during seed development [10,47,48]. The action of DOG1 in regulating PD is ABA-coordinated [10]. That is, the regulation of PD by DOG1 requires a functional ABA signaling pathway [1]. Thus, although *dog1* dormancy phenotypes are analogous to ABA synthesis and signaling mutants (i.e., ABA sensitivity of *dog1* seeds is unchanged [10,12,42]), prevailing evidences suggest that DOG1 and ABA act in independent pathways. That is, the *dog1* mutant is completely non-dormant and lacks apparent pleiotropic phenotypes, indicating that DOG1 is a key player specific for the induction of PD. Interestingly, the integration of the near isogenic line NILDOG1-Cvi (i.e., strong *DOG1* expression) with the ABA-deficient mutant *aba1-1* (i.e., impaired in ABA biosynthesis and absence of seed dormancy), still results in the production of non-dormant seeds [9]. This finding suggests that ABA is indispensable for the DOG1 function in seed dormancy, although it is fair to remind that ABA cannot impose seed dormancy in the absence of DOG1 [9]. Together, ABA and DOG1 must be present to induce PD as absence of one of these two regulators results in complete lack of PD even when the other regulator is highly accumulated [9,12,18]. Likewise, different groups demonstrated that *AtDOG1* is also crucial for local adjustment to distinct environments [19]. Therefore, *DOG1* is unquestionably related to the natural alteration of PD in *A. thaliana*; but this has not yet been proven in other species [13]. Finally, the correlation between DOG1 and ABA metabolism, transport, and signaling are not properly known. This study deserves special attention.

### 3.2. ABI3, ABI5, and DOG1

*ABI3* and *ABI5* encode essential ABA-dependent TFs and are two major components in the seed ABA signaling. DOG1 appears to control seed maturation and PD by controlling the expression of ABI5 (ABA INSENSITIVE 5; basic leucine zipper TF that functions in the core ABA signaling) and through genetic interaction with ABI3. These and other findings confirm that DOG1 is involved in the regulation of final phase of seeds development in coordination with ABA [9,13,23,25] (Figure 3). *ABI5* was not enough to suppress germination when overexpressed in Arabidopsis seeds and *abi5* shows a normal PD level [23]. Recent studies proposed that ABI5 is related to PD, and DOG1 regulates PD by controlling the *ABI5* expression [6]. Moreover, it was suggested that DOG1 stimulates ABI5 through the activation of seed maturation genes and the inhibition of germination-related transcripts [22]. Dekkers et al. (2016) [23] demonstrated that DOG1 interacts with PP2Cs, thus stimulating the expression of *ABI5* and PD. That is, the accumulation of ABA and ABI5 activity contributes to PD maintenance. In a nutshell, DOG1 regulates seed maturation and PD in part by controlling *ABI5* expression [23].

On the other hand, to determine the role of DOG1 in seed imbibition, several mutants related to the hormones synthesis and signaling have been analyzed, providing divergent results. For example, the ABA catabolic mutant *cyp77a2* exhibits high levels of ABA not only in dry seeds but also in seed imbibition, being highly dormant [49]. Nevertheless, the double mutant *dog1 cyp707a2* is less dormant than c*yp707a2* mutants, suggesting that the action of DOG1 is not completely ABA-dependent [10]. The Bentsink’s group [50] described that the *dog1* mutants exhibit normal response to exogenous ABA, proposing that DOG1 is unable to control ABA signaling during seed imbibition. On the other hand, *dog1-5* mutant has very strong PD and increased expression of miRNA processing genes [22]. Studies with *dog1-5* mutant indicated that the short *DOG1*-mRNA is subject to alternative polyadenylation and that the short form of this transcript is functional. Likewise, this short DOG1 protein isoform is a key player to PD establishment [3]. It is interesting to note that in *abi5* mutants, the expression of *DOG1* was upregulated in the presence of ABA. This suggests a crosstalk between *DOG1* expression and ABA responses during seed imbibition [51]. Likewise, several authors have considered the positive effect of ABA on the *DOG1* expression during germination of many species [52]. In *L. sativum* seed germination, ABA increased *LesaDOG*1-mRNA, possibly involving ABI3 and ABI5 [11]. Similarly, in cruciferae *Sysimbrium officinale,* ABA shows a positive effect on the *SoDOG1* expression during early imbibition. Moreover, this positive effect may be inhibited by optimal AR whereas GAs can partly mimic the AR effect [7]. Experiments of chromatin immunoprecipitation (ChIP) showed no direct interaction of ABI3 with the promoter region of *DOG1* [53]. However, some relation seems to exist between *DOG1* and ABI3 as the double mutant *abi3-1 dog1-1* shows a severe *abi3* phenotype that is not present in the single *abi3-1* mutant [23]. This genetic interaction could be explained by ABI3 acting downstream of *DOG1*, rather than upstream, with the ABA probably involved. On the contrary, FUS3 ChIP-chip analysis revealed a direct interaction with *DOG1*, presumably involving the RY-element (CATGCATG) present in its promoter region [53,54] (Figure 3). Microarray experiments showed that FUS3 knockout mutants in Arabidopsis have a significant decrease in *DOG1* expression [55].

Within the puzzle that constitutes the functioning of DOG1, the possibility of acting as a TF is currently being evaluated [32]. But there is still no scientific evidence that DOG1 works as a TF. The possibility emerges from the analysis of the amino acid sequence of DOG1. Thus, the DOG1 family share sequence similarity with the TGACG motif-binding TFs (TGAs) and may have evolved from them, or vice versa. It is striking that all TGAs of bZIP TFs contain the DOG1 superfamily domain. However, TGA task in seed development is not clear. Quite recently, DOGL4 was isolated from seeds and some properties are already known [32]. For example, DOGL4 lacks a bZIP domain. In [32] it is suggested that DOGL4 (and possibly DOGL5) is the missing link between the DOG1 family proteins and TGAs [32]. Thus, (i) unlike DOG1, DOGL4 is induced by ABA; (ii) DOGL4 shares scarce homology in amino acid sequence with DOG1; (iii) DOGL4 plays a major role in mediating SSPs accumulation in seeds; that is, DOGL4 is an inducer of SSPs; (iv) *dog1* does not alter the majority of DOGL4-induced SSPs; and (v) seeds from *dogl4* mutant exhibit moderately enhanced PD. Current knowledge of DOGL4 and DOG1 suggests that at a time of evolution some of the properties of these two members of the DOG1 family may have diverged into two independent seed maturation regulators for distinct biochemical functions [32]. In other words, taken together with the knowledge about DOGL4, it can be suggested that DOG1 family proteins may have first evolved as seed maturation regulators. Finally, the induction of the seed maturation genes by DOG1 and DOGL4 may not be mediated through direct DNA binding, but probably represents indirect regulation because DOG1 is a heme-binding protein [25,32,42].

### 3.3. Ethylene and DOG1

The interaction of ET with other phytohormones and ROS in the seed life regulation is well documented [4,56]. Thus, in the same way as GAs and ABA, the crucial control of ET traffic in the cell and their coordination in specialized seed tissues is of considerable importance during PD and germination. Recently, Li et al. (2019) identified a module regulating PD in Arabidopsis [44]. This group demonstrated that DOG1 functions downstream of both ET receptor 1 (ETR1; RDO3) and ET-responsive transcription factor-12 (ERF12). Likewise, they also demonstrate that *RDO3* encodes *ETR1*, an ET receptor. In fact, ERF12 binds directly to the DOG1 promoter, recruiting the co-repressor TOPLESS (TPL) in this nuclear process and inhibiting DOG1 expression. Likewise, through genetic analysis (i.e., *tpl-9 dog1-2* double mutant) this robust work insinuates that the regulation of PD by TPL depends on DOG1 [55]. In addition, (i) the transcriptional repressor in *A. thaliana* ERF12 (a member of the ERF-1B family) and TPL promote seed germination by repressing the DOG1 pathway; (ii) ERF12, functioning downstream of ETR1, is involved in regulating PD mediated by RDO3; (iii) ETR1 and ERF12 likely regulate PD through the DOG1 pathway [44]; and (iv) ERF12 binds to the promoter of *DOG1* and suppresses its expression (Figure 3). Together, perhaps DOG1 partially takes part in regulating PD mediated by the ET pathway. This possibility requires even more study. However, the role of the ETR1/RDO3-ERF12-TPL-DOG1 module discovered by the Li’s group has clarified a notable part of the ET-controlled PD mechanism.

## 4. Transcription Factors Directly Involved in DOG1 Task

Although knowledge of DOG1 has advanced considerably in recent years, it is not known meticulously which TFs bind to the DOG1 promoter and are responsible for driving its expression during embryo maturation. Since the amount of DOG1 accumulated in the dry seed determines the storage time necessary to release PD, the regulation of *DOG1* transcriptional activity by ABI3, FUS3, LEC1, and LEC2 is an exploration that should be considered in detail. *DOG1* expression is known to rely indirectly on LEC1, a member of the HAP3 family [45,57,58]. However, LEC1 has no direct interaction with *DOG1* promoter (Figure 3). In *lec1* mutants, the gene activity of *DOG1* is reduced. ABI3, FUS3, and LEC2 (known as AFL as a whole) contain a B3 DNA-binding domain (special to plants) that specifically recognizes RY [CATGCA(TG)] motif present in the promoter region of many maturation-related genes [15,45,57]. A RY motif is present in the promoter of *AtDOG1*, and its transcriptional pattern in seed development indicates that *DOG1* is very likely regulated by at least one of above TFs [10]. In the attempt to identify some TF with affinity for the DOG1 promoter, it was perceived in soybean (*Glycine max*) that bZIP67 is a regulator of several genes involved in SSPs deposition [59]. In other words, *DOG1* encodes a plant specific protein with a domain shared by bZIP. Progressing in this approach, quite recently the Eastmond’s group proved in a solid work that bZIP67 acts downstream LEC1 to transactivate DOG1 during seed maturation helping to establish PD in Arabidopsis [60]. Eastmond in his work demonstrated that: (i) bZIP67 is required for *DOG1* expression and DOG1 accumulation; (ii) probably, bZIP67 and DOG1 functionally belong to same pathway; (iii) bZIP67 may contribute to the regulation of PD through the control of *DOG1* expression; (iv) DOG1 is induced by LEC1; this fact occurs after the induction of bZIP67; (v) in vivo and in vitro experiments propose that bZIP67 binds to the promotor of DOG1 by GBL *cis*-element; (vi) cool conditions during seed maturation enhances bZIP67 amount but not bZIP67-mRNA [60]. Together, bZIP67 is a direct regulator of DOG1 expression, specifying the LEC1 action in the establishment of PD (Figure 3).

## 5. The Relationship between DOG1 and Protein Phosphatases

The major advances to unravel the molecular function of DOG1 are probably those provided by recent studies of direct interaction with other proteins. Pull-down experiments in vivo carried out by Née et al. [25] revealed 184 groups of proteins with direct interaction with DOG1. These results confirm again that DOG1 interacts with proteins involved in ABA responses [20,25]. Among these pulled down proteins there are some members of PP2C family [25,42]. PP2C phosphatases act as negative regulators of ABA signaling by inactivating sucrose nonfermenting-I-related protein kinases-2 (SnRK2), that act as positive effectors of ABA response. ABA receptors PYrabactin resistance-1 (PYR1)/PYR1-Like (PYL)/regulatory component of ABA receptor (RCAR) inactivate PP2Cs in the presence of ABA, allowing SnRK2s to trigger ABA-associated responses [48]. Two subfamilies of the PP2Cs (group-A) have been described in Arabidopsis: ABI1 and ABA-hypersensitive germination-1 (AHG1) [61]. The ABA receptors PYR1 interact with ABI1 subfamily members in the presence of ABA but not with AHG1 subfamily members, except for AHG3. On the contrary, DOG1 interacts with the AHG1 subfamily members but not with the ABI1 subfamily members [42]. These data suggest that PP2Cs could be the connection point between ABA and DOG1 signaling pathways. In addition to physically interacting, it has been demonstrated that *AHG1* and *AHG3* (i.e., both expressed in seeds with AHG1 being more specific to seeds than AHG3) are necessary for DOG1 functioning. Thus, while double mutant (*dog1-ahg1* and *dog1-ahg3)* show the same non-dormant phenotype than *dog1*, triple mutant (*dog1-ahg1-ahg3)* show highly dormant phenotype [25]. These data suggest that AHG1 and AHG3 act redundantly as negative regulators of PD induction by DOG1. The relationship between DOG1 and AHG1 has been studied in detail. Thus, it seems clear that DOG1 functions upstream of AHG1 in the ABA signaling pathway, and directly regulates the PP2C activity of AHG1 in an ABA-dependent manner [25]. Unlike AHG3, AHG1 does not interact with ABA receptors. In Arabidopsis, the deletion of residues 1-18 in the N-terminal region of DOG1 protein has a strong negative effect on both the interaction with AHG1 and the induction of PD [42]. This deletion prevents the ability of DOG1 to confer an ABA-hypersensitive phenotype, indicating that the interaction with AHG1 is indispensable for DOG1 function in both PD and germination control. Curiously, this small N-terminal region is not especially well conserved among species and it is unknown whether its deletion may affect the DOG1 conformation or only its interaction with AGH1 [14]. On the contrary, the deletion of 257-291 residues in the C-terminal region does not affect the interaction of DOG1 with AHG1 [42]. Very recently, the Nishimura’s group demonstrates that the DSYLEW N-terminal sequence of DOG1 (spanning position 13–18), is essential for interacting with AHG1 [42]. Taken together, DOG1 enhances ABA signaling through its binding to AHG1 and AHG3 [25].

The presence of DOG1 decreases the AHG1 activity on its target SnRK2s. As in the case of PYL/PYR/RCAR, this indicates that DOG1 positively regulates the SnRK2s activity by inhibiting the PP2C activity of AHG1 [42]. It is likely that DOG1 has an effect on AHG3, similarly to the observed in AHG1, but until now the activity assays were carried out using artificial substrates for PP2C and it would be necessary to analyze its phosphatase activity specifically on SnRK2s [25,42]. So far, the existing data suggest that, (i) ABI1 phosphatase subfamily is regulated by PYR/PYL/RCAR receptor in presence of ABA; (ii) AHG1 is regulated by DOG1; and (iii) AHG3 is regulated by both ABA and DOG1. These interactions could explain the partial overlap between ABA and DOG1 mechanisms of action. At the same time, they could answer to the dormancy phenotype observed in *dog1* and ABA biosynthesis mutants [10]. In mutants with low expression of *DOG1*, the presence of ABA may not regulate the AHG1 activity, which continues acting as negative regulator of PD. On the contrary, in mutants with low ABA content, members of PP2C subfamily ABI1 remain active and would negatively regulate PD levels [42]. In addition to dephosphorylating SnRK2s, AHG1 also interacts with other proteins [42]. One of them is ABI FIVE BINDING PROTEIN 2 (AFP2), that acts as a negative regulator of the ABA response and interacts with ABI5 promoting its degradation [62]. These interactions of AHG1 could be important in the regulatory role of DOG1 too.

DOG1 has also shown to interact with reduced dormancy 5 (RDO5), a promoter of Arabidopsis PD, which belongs to PP2C family; but it lacks phosphatase activity. Curiously, mutant *rdo5* shows a reduced PD level, contrary to what occurs in mutant *ahg1-ahg3* [27,63]. If this pseudo phosphatase is involved in DOG1 signaling, its function would probably be different from other PP2Cs. It was demonstrated that RDO5 influences the PD analogously to DOG1 and, moreover, it mainly appears to function independent of ABA [63]. *RDO5* and *DOG1* have some similarities: (i) Seed preferential expression; (ii) positive regulators of PD; and (iii) their mutants exclusively show dormancy and germination defects without pleiotropic phenotypes [64]. The molecular mechanisms used by these dormancy-specific genes for regulating PD and their relationship with ABA signaling is still unclear and need to be studied more carefully [63]. Additionally, DOG1 also interacts with PROTEIN PHOSPHATASE 2A SUBUNIT A2 (i.e., PP2AA/PDF1) that acts as a negative regulator of PD. PDF1 acts upstream of DOG1 probably dephosphorylating DOG1 during the seed imbibition [27,65]. Concluding, all recent genetic analysis of this section indicate that: (i) The interaction of DOG1 with AHG1, AHG3, and RDO5 [25,42] was observed in the nucleus, whereas that the interaction with PDF1 (a DOG1 interacting phosphatase) occurs in the cytosol [25]; (ii) genetic analysis suggests that DOG1 acts as a suppressor of AHG1 and AHG3 action in PD release; (iii) AHG1 and AHG3 have redundant roles in seed dormancy because the phenotype of the double mutant is much more severe than that of the single mutants; (iv) similar to *dog1-2*, the *pdf1* and *dog1-2* double mutant completely lacked PD. Therefore, *dog1-2* appears to be epistatic to *pdf1*, suggesting that DOG1 functions downstream of PDF1; and (v) ABA and DOG1 pathways converge at the level of the clade-A PP2C [20]. Interestingly, the loss of RDO5 function could be compensated for by low temperatures [20].

## 6. DOG1 also Undergoes Epigenetic Regulation

Chromatin modifications (e.g., methylation) are involved in the PD regulation; but little is known about its esential mechanism. It is interesting to highlight that methylation of histone 3 at Lys-4 (i.e., H3K4me3; active chromatin) in *DOG1* is more abundant in dormant seeds; while the repressing chromatin H3K27me3 predominates in germinating seeds. The polycomb repressing complex 1 (PRC1) seems to play a key role in these methylation changes. PRC1 interacts with the H3K4me3-binding ALFIN-like proteins and recruits PRC2 that establishes H3K27me3 marks [66]. In addition to the studies on *as*RNA and the reciprocal regulation of *DOG1*-*asDOG1* pair [16,67], the TFs HISTONE MONOUBIQUITYLATION-1 (HUB1), and REDUCED DORMANCY-2 (RDO2), involved in chromatin compaction, are required for the establishment of PD in *A. thaliana*. HUB1 and RDO2 are upregulated during seed maturation [68,69]. The *hub1* mutant have reduced seed dormancy [68]. Transcriptomic analysis of *hub1* (*rdo4*) and *rdo2* revealed a reduction of *DOG1* expression that could be associated to diminished PD [70]. Interestingly, PD level of *rdo2* increases whereas ABA content and sensitivity remain unchanged [4,63]. Moreover, *hub1* mutants obtained in a Landsberg *erecta* (Ler-0) accession were crossed with the near isogenic line NILDOG1 (i.e., strong DOG1 expression). The seeds from *hub1* mutants containing the DOG1-Cvi introgression were more dormant than those obtained from the original *hub1* mutant in a simple Ler background. The main conclusion of this experiment could be that *HUB1* is not epistatic to *DOG1* [68]. Therefore, *HUB1* is probably acting upstream of *DOG1* in combination with other regulatory mechanisms. In *rdo2* mutants, the addition of an extra copy of *DOG1* reversed the reduced PD back to wild type levels. The reduction of *DOG1* expression in this experiment is, at least in part, responsible for the dormancy phenotype observed in *rdo2* mutants [69,71]. Despite the above mentioned, *DOG1* is not necessarily regulated by HUB1 and RDO2 in a direct path. Many genes associated with maturation are also affected in *hub1* and *rdo2* mutants, including many genes associated with hormone metabolism and PD establishment, which can be mediating in the reduction of *DOG1* expression [69]. Contrary to *HUB1* and *RDO1*, the *KRYPTONITE/SUVH4* (*KYP*) gene encodes a methyltransferase involved in the regulation of PD acting as a negative regulator of the transcription processes [72,73]. *HUB* and *RDO1* are epistatic with respect to *KYP* [73]. Overexpression and mutations in *KYP* causes decreased and increased PD, respectively [73]. *KYP* gene is involved in the regulation of both ABA and GAs sensitivity in the seed and influences the transcriptional activity of DOG1 and other PD-related genes (e.g., *DOG1* and *ABI3*). Alternatively, KYP function could also be mediated by the alterations in the ABA: GAs balance rather than by an alteration of DOG1 histone methylation status. Interestingly, the analysis of double mutants *kyp-dog1*, *kyp-hub1,* and *kyp-rdo2* suggest that HUB1 and KYP act in the same pathway to regulate DOG1, while RDO2 acts in a parallel path [73]. In general, *HUB1* expression rises previously to the induction of PD, overlapping with an increase of *DOG1* transcription. It is striking that *KYP* expression is maximum in the summer when the PD and *DOG1* expression levels are low [32].

## 7. Are the Post-Translational Modifications of DOG1 Involved in Seed After-Ripening?

The PD can be relieved through a highly regulated process called AR that occurs in the dry viable seed [35,74,75]. AR upregulates the *sensu-stricto* germination modulating the sensitivity and metabolism of ABA [35,75]. The DOG1 protein becomes modified during AR, and its levels in stored viable seeds do not correlate with germination potential [10,12]. These protein modifications might prevent or reduce DOG1 self-binding and thereby its function [26]. The intensity of PD is directly related to DOG1 protein level accumulated in the dry mature viable seeds. This level defines the time that the seed must be stored to produce the PD release. DOG1 expression is associated with dormancy variation measured as AR time [9,26]. As a rule, the large amount of DOG1-mRNA accumulated in deep dormant seeds decreases during the onset of imbibition of both dormant and non-dormant seeds, but this decrease is faster in AR and germinating seeds [7,10]. Interestingly, the loss of PD by AR is not associated with an important reduction of the protein levels in the seed, but it could be related to a decrease of the DOG1 activity mediated by not yet known post-translational modifications [35,76]. Recently, it was proven that exogenous ABA increases the SoDOG1-mRNA levels. However, this increase is downregulated when optimal AR has been already established [7]. The inactivation of DOG1 might operate as a timer for overcoming PD and take part in a mechanism for controlling PD release [10,20]. Nakabayashi et al. (2012) proposed that post-translational protein modification (i.e., a shift in the isoelectric point; pI) makes DOG1 non-functional (i.e., inactivation) prior to and following AR [10]. These authors suggest a change in DOG1 structure and loss of function caused by AR. PDF1, a DOG1 interacting phosphatase, acts as a negative regulator of PD and its mutation prevents the isoelectrofocusing change of DOG1 during imbibition [10,25]. However, the germination of *pdf1* mutants is only slightly increased, and therefore other post-translational regulatory mechanisms may be necessary for the inactivation of DOG1. It is unknown how this process takes place, but it could be caused by a non-enzymatic oxidative process since, as mentioned above, they seem to have great importance in seed AR [43,75,77]. Consequently, the modifications experienced by DOG1 in the course of AR and the loss of PD might be due to oxidative processes [10]. In order to prove it, in a QTL mapping following the elevated partial pressure of oxygen (EPPO) treatment, *DOG1, DOG2,* and *DOG6* loci were identified. These loci had been previously identified employing AR to overcome PD [20,49]. As a conclusion to this approach it can be suggested that the release of PD by AR is principally produced by oxidative processes, and oxidative post-translational modifications of DOG1 could probably be associated with AR [20,65].

As indicated above, there is a negative correlation between the potential of germination and DOG1 protein levels [10,12]. But there are two important exclusions. On the one hand, the AR seeds may germinate even in high levels of DOG1 protein [10]. On the other hand, Nakabayashi et al. [26] showed that some seeds might germinate if they possess DOG1 unable to bind itself. In both cases, the absence of a negative correlation between the potential of germination and the accumulation of DOG1 protein can be due to similar causes. Accordingly, it is feasible that AR is related to the modifications of DOG1 protein which could affect its function, probably by avoiding or decreasing DOG1 self-binding [26]. Nevertheless, it is necessary to consider that DOG1 might act in PD signaling by an alternative pathway than in AR since it was proved that PD and AR seem to be independent at the molecular level [78]. Analysis of fully AR seeds in the Columbia (Col) background showed a decrease in ABA sensitivity for the *dog1-2* mutant [25].

## 8. Perspectives for Future Research on DOG1

Throughout this study we have reviewed a good number of recent publications related to DOG1, a protein absolutely required for the induction of PD. However, its molecular function is largely unknown. The hypothesis and conclusions that are currently underway in this review are largely based on data obtained from *A. thaliana* seed mutants and the characterization of cofactors that bind to different sites of the DOG1 structure. All known genetic and molecular data are part of a highly complex puzzle that, acting in a coordinated mode with ABA, trigger the generation of active DOG1 and appearance of PD. However, although the function of DOG1 seems conserved in Angiosperms, much more experimentation in monocot and dicot species needs to be done to confirm. The key to addressing the emergence and evolution of DOG1 involves studying similar genes in the DOG1 family (see Figure 1). Given the gaps existing in the knowledge of the mechanism of action of ABA in the PD process, this evolutionary study of DOG1 is of enormous importance since it firmly proves that DOG1 inhibits germination in an ABA-dependent way. However, ABA accumulates in seeds mainly to prevent viviparism and establish PD. The relationship between DOG1 and development of viviparism will be an important applied approach because reduced yield of certain crops is involved. On the other hand, the knowledge of endogenous and environmental signals responsible for the appearance/disappearance of DOG1 in the cells involved in the triggering, maintenance, and loss of PD, is an aspect still obscure and that requires a great deal of dedication. Recent research found that the production of miRNAs others than miR156 and miR172 (i.e., miR319, miR160, miR164, and miR390) are affected by DOG1 in lettuce and Arabidopsis. Although it seems clear that some miRNAs affect DOG1, it has not been conclusively proven yet that miRNA intervention is part of the primary mechanism by which DOG1 regulates PD. Hence, this intriguing and novel aspect should be taken into consideration to demonstrate the function of DOGs more comprehensively.

**Figure 4:**
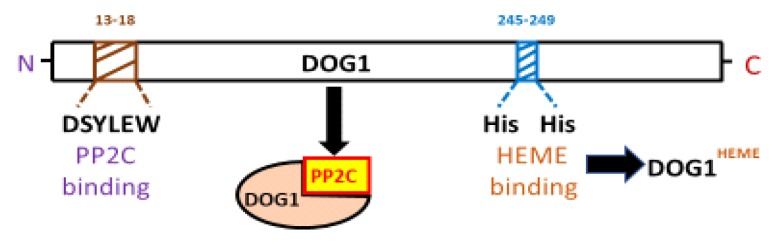
AtDOG1 protein structure indicating the positions of the AHG1 and heme binding sites. DOG1 is an α-helical protein that has the ability to bind both ABA HYPERSENSITIVE GERMINATION1 (AHG1; a clade A protein phosphatase 2C) and heme group. The amino acid residues that specifically bind PP2C (i.e., DSYLEW) are very close to the N-terminal; while those that specifically bind heme group are closer to the C-terminal. Heme binding is not necessary for DOG1 interaction with AHG1. However, heme binding at His245 and His249 is essential for DOG1 function in seed dormancy. Modified from Nonogaki (2019) [1,48].

**Figure 5:**
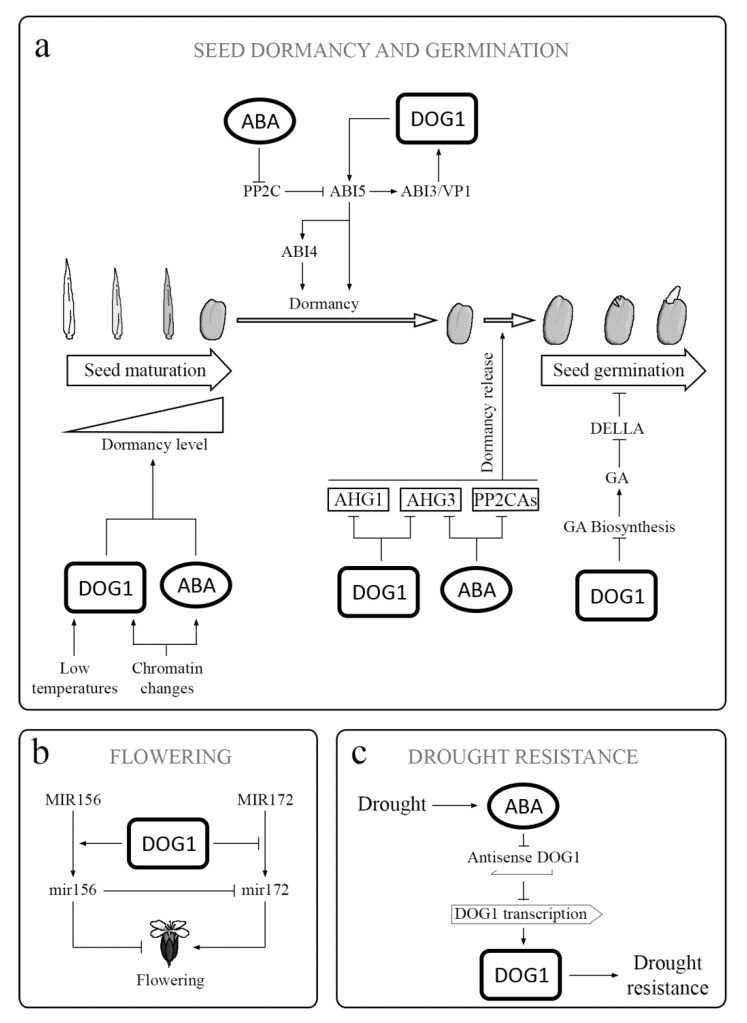
DOG1 in different physiological processes: (**a**) DOG1 has an influence on several genes involved in ABA signaling, including *ABI3* and ABI5. Thus, it acts together with ABA in the regulation of the acquisition, maintenance, and release of PD. DOG1 also inactivates AHG1 and AHG3, which are the key negative regulators in ABA signaling. The inactivation of AHG1 and AHG2 results in release of PD. Additionally, DOG1 reduces the GAs biosynthesis during germination. (**b**) DOG1 participates in the regulation of flowering by influencing the transition from primary MIR156 and MIR172 to active miR156 and miR172 through an effect on expression of genes involved in miRNA processing. Both active miRNAs are regulators, not only of flowering but also of PD and seed and seedling development. (**c**) Under drought conditions, the ABA content increases. The expression of DOG1 enhances the drought tolerance in vegetative tissues, and asDOG1 transcription is ABA repressed. asDOG1 acts as a negative regulator of DOG1 transcription.

The yeast two-hybrid analysis (YTHA), co-immunoprecipitation, plant co-transformation, and epistatic analysis, are essential tools to progress in the knowledge of DOG1 at the molecular level. As a proof of the use of these methodologies, the breakthrough came from recent investigation about DOG1-interacting proteins (e.g., AHG1-DOG1). Although the interaction of DOG1 with AHG1 seems demonstrated, it is still not clear if the inactivation of AHG1 by DOG1 in vivo is a consequence of that interaction. Given that DOG1 affects the expression level of *ABI5*, the DOG1-AHG1 complex likely regulates ABI5 function. But this fact is still unsolved. Intriguingly, since the absorption spectrum of the active DOG1 exhibits characteristics of a heme-protein complex, it was proven that DOG1 binds to a heme group using His residues from its protein (Figure 4). It is striking that heme is not fundamental for DOG1 to interact with AHG1. Once it was known that heme group is involved in the action of DOG1, the PD mechanisms goes through a thorough investigation into the DOG1-heme relationship. Parallelly, at the evolutionary level, more research on ABA and heme signaling in the algal ancestor and bryophytes, are essential. However, the origin of heme in seeds is still an open question. Thus, is the plasmid only the source of heme in the seed cells or is there heme of mitochondrial origin?. This question opens an interesting line of research since the heme group has a remarkable interference in many aspects of seed physiology. Further studies are needed to elucidate the potential role of heme bound to DOG1 as a sensor of O_2_ or NO. Thus, in the course of AR, several oxidative processes are produced, causing post-translational modifications in DOG1. It is necessary to investigate the possibility that ROS could oxidize DOG1. The nature of DOG1 modifications (e.g., phosphorylation/de-phosphorylation, redox changes, chromatin alterations, etc.,) in dry or imbibed AR viable seeds has not been demonstrated explicitly. These modifications may contribute to the change in the configuration of the DOG1 protein. Therefore, to carry out this approach, the study of the AR process in mutant seeds that are affected in the functioning of DOG1 can be an interesting tool. Finally, the knowledge in depth of PD physiology will imply to have tools for the control, among others, of crops with high agricultural value. In other words, the identification of DOG1 protein modifications is key to our understanding of PD and could enable the manipulation of PD levels in crop plants. Thus, a suitable understanding of PD will be advantageous for both ecological understanding and crop management. Likewise, it will be interesting to find out whether DOG1 is involved in the adaptation of PD to other environmental conditions that occur during seed maturation, like drought (Figure 5), light intensity, daylength, high or low temperatures, and nitrate levels. Together, the knowledge in depth of PD physiology will imply to have tools for the control, among others, of crops with high agricultural interest. Finally, to highlight the CRISPR/Cas (clustered regularly interspaced short palindromic repeats/CRISPR-associated protein) based genome editing approach has become a choice of technique due to its simplicity, ease of access, and flexibility [79,80]. The CRISPR/Cas9 system is a revolutionary technology for crop breeding and biological research through direct and controlled changes in the genome. Thus, the potential to edit multiple targets simultaneously makes CRIPSR/Cas9 possible to take up more challenging tasks required to engineer desired crop plants. The new gene editing techniques are more precise than standard genetic engineering tools that have been previously developed. The use of this technology will cooperate in the strong advance of knowledge of DOG1.

## Supplementary Materials

The following are available online at https://www.mdpi.com/2223-7747/9/4/480/s1, Figure S1: Alignment of deduced DOG1 and DOG1-like amino acid sequences used to create the phylogenetic tree (Figure 1). Sequences were aligned using MEGA-X software and the Clustal-W algorithm, Table S1: Genes and proteins included in the phylogenetic tree.

## References

[1] Nonogaki H. (2019). Seed germination and dormancy: The classic story, new puzzles, and evolution. J. Integr. Plant Biol..

[2] Graeber K., Voegele A., Büttner-Mainik A., Sperber K., Mummenhoff K., Leubner-Metzger G. (2013). Spatiotemporal seed development analysis provides insight into primary dormancy induction and evolution of the *Lepidium DELAY OF GERMINATION 1* genes. Plant Physiol..

[3] Cyrek M., Fedak H., Ciesielski A., Guo Y., Sliwa A., Brzezniak L., Krzyczmonik K., Pietras Z., Kaczanowski S., Liu F. (2016). Seed dormancy in Arabidopsis is controlled by alternative polyadenylation of *DOG1*. Plant Physiol..

[4] Shu K., Liu X.D., Xie Q., He Z.H. (2016). Two faces of one seed: Hormonal regulation of dormancy and germination. Mol. Plant.

[5] Ashikawa I., Mori M., Nakamura S., Abe F.A. (2014). A transgenic approach to controlling wheat seed dormancy level by using Triticeae *DOG1*-like genes. Transgenic Res..

[6] Zhao M., Yang S., Liu X., Wu K. (2015). Arabidopsis histone demethylases LDL1 and LDL2 control primary seed dormancy by regulating *DELAY OF GERMINATION 1* and ABA signaling-related genes. Front. Plant Sci..

[7] Carrillo-Barral N., Matilla A.J., García-Ramas C., Rodríguez-Gacio M.C. (2015). ABA-stimulated *SoDOG1* expression is after-ripening inhibited during early imbibition of germinating *Sisymbrium officinale* seeds. Physiol. Plant..

[8] Alonso-Blanco C., Bentsink L., Hanhart C.J., Blankestijn-de Vries H., Koornneef M. (2003). Analysis of natural allelic variation at seed dormancy loci of *Arabidopsis thaliana*. Genetics.

[9] Bentsink L., Jowett J., Hanhart C.J., Koornneef M. (2006). Cloning of DOG1, a quantitative trait locus controlling seed dormancy in Arabidopsis. Proc. Natl. Acad. Sci. USA.

[10] Nakabayashi K., Bartsch M., Xiang Y., Miatton E., Pellengahr S., Yano R., Seo M., Soppe W.J.J. (2012). The time required for dormancy release in *Arabidopsis* is determined by DELAY OF GERMINATION1 protein levels in freshly harvested seeds. Plant Cell.

[11] Graeber K., Linkies A., Müller K., Wunchova A., Rott A., Leubner-Metzger G. (2010). Cross-species approaches to seed dormancy and germination: Conservation and biodiversity of ABA-regulated mechanisms and the Brassicaceae *DOG1* genes. Plant Mol. Biol..

[12] Graeber K., Linkies A., Steinbrecher T., Mummenhoff K., Tarkowska D., Tureckova V., Ignatz M., Sperber K., Voegele A., de Jong H. (2014). *DELAY OF GERMINATION 1* mediates a conserved coat-dormancy mechanism for the temperature-and gibberellin-dependent control of seed germination. Proc. Natl. Acad. Sci. USA.

[13] Huo H., Wei S., Bradford K.J. (2016). DELAY OF GERMINATION1(DOG1) regulates both seed dormancy and flowering time through microRNA pathways. Proc. Natl. Acad. Sci. USA.

[14] Ashikawa A., Abe F., Nakamura S. (2013). *DOG1*-like genes in cereals: Investigation of their function by means of ectopic expression in Arabidopsis. Plant Sci..

[15] Rikiishi K., Maekawa M. (2014). Seed maturation regulators are related to the control of seed dormancy in wheat (*Triticum aestivum* L.). PLoS ONE.

[16] Fedak H., Palusinska M., Krzyczmonik K., Brzezniak L., Yatusevich R., Pietras Z., Kaczanowski S., Swiezewski S. (2016). Control of seed dormancy in Arabidopsis by a cis-acting noncoding antisense transcript. Proc. Natl. Acad. Sci. USA.

[17] Chiang G.C.K., Bartsch M., Barua D., Nakabayashi K., Debieu M., Kronholm I., Koornneef M., Soppe W.J.J., Donohue K., De Meaux J. (2011). *DOG1* expression is predicted by the seed-maturation environment and contributes to geographical variation in germination in Arabidopsis thaliana. Mol. Ecol..

[18] Kendall S.L., Hellwege A., Marriot P., Whalley C., Graham I.A., Penfield S. (2011). Induction of dormancy in *Arabidopsis* summer annuals requires parallel regulation of *DOG1* and hormone metabolism by low temperature and CBF transcription factors. Plant Cell..

[19] Murphey M., Kovach K., Elnaccash T., He H., Bentskink L., Donohue K. (2015). DOG1-imposed dormancy mediates germination responses to temperature cues. Environ. Exp. Bot..

[20] Née G., Xiang Y., Soppe W.J. (2017). The release of dormancy, a wake-up call for seeds to germinate. Curr. Opin. Plant Biol..

[21] Buijs G., Kodde J., Groot S.P.C., Bentsink L. (2018). Seed dormancy release accelerated by elevated partial pressure of oxygen is associated with DOG loci. J. Exp. Bot..

[22] Yatusevich R., Fedak H., Ciesielski A., Krzyczmonik K., Kulik A., Dobrowolska G., Swiezewski S. (2017). Antisense transcription represses *Arabidopsis* seed dormancy QTL *DOG1* to regulate drought tolerance. EMBO Rep..

[23] Dekkers B.J., He H., Hanson J., Willems L.A., Jamar D.C., Cueff G., Rajjou L., Hilhorst H.W., Bentsink L. (2016). The Arabidopsis DELAY OF GERMINATION 1 gene affects ABSCISIC ACID INSENSITIVE 5 (ABI5) expression and genetically interacts with ABI3 during Arabidopsis seed development. Plant J..

[24] Auge G.A., Penfield S., Donohue K. (2019). Pleiotropy in developmental regulation by flowering-pathway genes: Is it an evolutionary constraint?. New Phytol..

[25] Née G., Kramer K., Nakabayashi K., Yuan B., Xiang Y., Miatton E., Finkemeier I., Soppe W.J.J. (2017). DELAY OF GERMINATION1 requires PP2C phosphatases of the ABA signaling pathway to control seed dormancy. Nat. Commun..

[26] Nakabayashi K., Bartsch M., Ding J., Soppe W.J.J. (2015). Seed dormancy in Arabidopsis requires self-binding ability of DOG1 protein and the presence of multiple isoforms generated by alternative splicing. PLoS Genet..

[27] Gutierrez L., Van Wuytswinkel O., Castelain M., Bellini C. (2007). Combined networks regulating seed maturation. Trends Plant Sci..

[28] Bewley J.D., Nonogaki H. (2017). Seed Maturation and Germination. Reference Module in Life Sciences.

[29] Zinsmeister J., Lalanne D., Terrasson E., Chatelain E., Vandecasteele C., Vu B.L., Dubois-Laurent C., Geoffriau E., Le Signor C., Dalmais M. (2016). ABI5 is a regulator of seed maturation and longevity in legumes. Plant Cell.

[30] Leprince O., Pellizzaro A., Berriri S., Buitink J. (2017). Late seed maturation: Drying without dying. J. Exp. Bot..

[31] Li P., Ni H., Ying S., Wei J., Hu X. (2020). Teaching an old dog a new trick: multifaceted strategies to control primary seed germination by DELAY OF GERMINATION 1 (DOG1). Phyton-Int. J. Exp. Bot..

[32] Sall K., Dekkers B.J.W., Nonogaki M., Katsuragawa Y., Koyari R., Hendrix D., Willems L.A.J., Bentsink L., Nonogaki H. (2019). DELAY OF GERMINATION 1-LIKE 4 acts as an inducer of seed reserve accumulation. Plant J..

[33] Seo M., Marion-Poll A. (2019). Abscisic acid metaboolism and transport. Adv. Bot. Res..

[34] Finch-Savage W.E., Footitt S. (2017). Seed dormancy cycling and the regulation of dormancy mechanisms to time germination in variable field environments. J. Exp. Bot..

[35] Matilla A.J., Carrillo-Barral N., Rodríguez-Gacio M.C. (2015). An Update on the Role of NCED and CYP707A ABA metabolism genes in seed dormancy induction and the response to after-ripening and nitrate. J. Plant Growth Regul..

[36] Footitt S., Walley P.G., Lynn J.R., Hambidge A.J., Penfield S., Finch-Savage W.E. (2020). Trait analysis reveals DOG1 determines initial depth of seed dormancy, but not changes during dormancy cycling that result in seedling emergence timing. New Phytol..

[37] Postma F.M., Lundemo S., Ågren J. (2016). Seed dormancy cycling and mortality differ between two locally adapted populations of *Arabidopsis thaliana*. Ann. Bot..

[38] Postma F.M., Agren J. (2016). Early life stages contribute strongly to local adaptation in *Arabidopsis thaliana*. Proc. Natl. Acad. Sci. USA.

[39] Née G., Obeng-Hinneh E., Sarvari P., Nakabayashi K., Soppe W.J.J. (2015). Secondary dormancy in *Brassica napus* is correlated with enhanced *BnaDOG1* transcript levels. Seed Sci. Res..

[40] Bazin J., Langlade N., Vincourt P., Arribat S., Balzergue S., El-Maarouf-Bouteau H., Bailly C. (2011). Targeted mRNA oxidation regulates sunflower seed dormancy alleviation during dry after-ripening. Plant Cell.

[41] Huang D., Koh C., Feurtado J.A., Tsang E.W., Cutler A.J. (2013). MicroRNAs and their putative targets in *Brassica napus* seed maturation. BMC Genom..

[42] Nishimura N., Tsuchiya W., Moresco J.J., Hayashi Y., Satoh K., Kaiwa N., Irisa T., Kinoshita T., Schroeder J.I., Yates J.R. (2018). Control of seed dormancy and germination by DOG1-AHG1 PP2C phosphatase complex via binding to heme. Nat. Commun..

[43] Bailly C. (2019). The signalling role of ROS in the regulation of seed germination and dormancy. Biochem. J..

[44] Li X., Chen T., Li Y., Wang Z., Cao H., Chen F., Li Y., Soppe W.J.J., Li W., Liu Y. (2019). ETR1/RDO3 regulates seed dormancy by relieving the inhibitory effect of the ERF12-TPL complex on DELAY OF GERMINATION1 expression. Plant Cell.

[45] Carbonero P., Iglesias-Fernández R., Vicente-Carbajosa J. (2017). The AFL subfamily of B3 transcription factors: Evolution and function in angiosperm seeds. J. Exp. Bot..

[46] Breeze E. (2019). Letting sleeping DOGs lie: Regulation of DOG1 during seed dormancy. Plant Cell..

[47] Szakonyi D., Duque P. (2018). Alternative splicing as a regulator of early plant development. Front. Plant Sci..

[48] Nonogaki H. (2019). ABA responses during seed development and germination. Adv. Bot. Res..

[49] Okamoto M., Kuwahara A., Seo M., Kushiro T., Asami T., Hirai N., Kamiya Y., Koshiba T., Nambara E. (2006). CYP707A1 and CYP707A2, which encode abscisic acid 8′-hydroxylases, are indispensable for proper control of seed dormancy and germination in Arabidopsis. Plant Physiol..

[50] Bentsink L., Hanson J., Hanhart C.J., de Vries H.B., Coltrane C., Keizer P., El-Lithy M., Alonso-Blanco C., de Andres M.T., Reymond M. (2010). Natural variation for seed dormancy in Arabidopsis is regulated by additive genetic and molecular pathways. Proc. Natl. Acad. Sci. USA.

[51] Kinoshita N., Berr A., Belin C., Chappuis R., Nishizawa N.K., Lopez-Molina L. (2010). Identification of growth insensitive to *ABA3* (*gia3*), a recessive mutation affecting ABA signaling for the control of early postgermination growth in *Arabidopsis thaliana*. Plant Cell Physiol..

[52] Teng S., Rognoni S., Bentsink L., Smeekens S. (2008). The Arabidopsis GSQ5/DOG1 Cvi allele is induced by the ABA-mediated sugar signalling pathway and enhances sugar sensitivity by stimulating ABI4 expression. Plant J..

[53] Mönke G., Altschmied L., Tewes A., Reidt W., Mock H.P., Bäumlein H., Conrad U. (2004). Seed-specific transcription factors ABI3 and FUS3: Molecular interaction with DNA. Planta.

[54] Wang F., Perry S.E. (2013). Identification of direct targets of FUSCA3, a key regulator of *Arabidopsis thaliana* seed development. Plant Physiol..

[55] Yamamoto A., Kagaya Y., Usui H., Hobo T., Takeda S., Hattori T. (2010). Diverse roles and mechanisms of gene regulation by the Arabidopsis seed maturation master regulator FUS3 revealed by microarray analysis. Plant Cell Physiol..

[56] Nonogaki H. (2017). Seed biology updates—Highlights and new discoveries in seed dormancy and germination research. Front Plant Sci..

[57] Giraudat J., Hauge B.M., Valon C., Smalle J., Parcy F., Goodman H.M. (1992). Isolation of the Arabidopsis *ABI3* gene by positional cloning. Plant Cell.

[58] Pelletier J.M., Kwong R.W., Park S., Le B.H., Baden R., Cagliari A., Hashimoto M., Muñoz M.D., Fischer F.L., Goldberg R.B. (2017). LEC1 sequentially regulates the transcription of genes involved in diverse developmental processes during seed development. Proc. Natl. Acad. Sci. USA.

[59] Dröge-Laser W., Snoek B.L., Snel B., Weiste C. (2018). The Arabidopsis bZIP transcription factor family—An update. Curr. Opin. Plant Biol..

[60] Bryant F.N., Hudges D., Hassani-Pak K., Eastmond P.J. (2019). Basic LEUCINE ZIPPER TRANSCRIPTION FACTOR67 transactivates DELAY OF GERMINATION1 to establish primary seed dormancy in Arabidopsis. Plant Cell.

[61] Xue T., Wang D., Zhang S., Ehlting J., Ni F., Jakab S., Zeng C., Zhong Y. (2008). Genome-wide and expression analysis of protein phosphatase 2C in rice and Arabidopsis. BMC Genomics.

[62] Garcia M.E., Lynch T., Peeters J., Snowden C., Finkelstein R. (2008). A small plant specific protein family of ABI five binding proteins (AFPs) regulates stress response in germinating Arabidopsis seeds and seedlings. Plant Mol. Biol..

[63] Xiang Y., Nakabayashi K., Ding J., He F., Bentsink L., Soppe W.J.J. (2014). Reduced dormancy 5 encodes a protein phosphatase 2C that is required for seed dormancy in *Arabidopsis*. Plant Cell.

[64] Kerdaffrec E., Filiault D.L., Korte A., Sasaki E., Nizhynska V., Seren Ü., Nordborg M. (2016). Multiple alleles at a single locus control seed dormancy in Swedish Arabidopsis. eLife.

[65] Zhou H.W., Nussbaumer C., Chao Y., DeLong A. (2004). Disparate roles for the regulatory A subunit isoforms in Arabidopsis protein phosphatase 2A. Plant Cell.

[66] Molitor A.M., Bu Z., Yu Y., Shen W.H. (2014). Arabidopsis AL PHD-PRC1 complexes promote seed germination through H3K4me3-to-H3K27me3 chromatin state switch in repression of seed developmental genes. PLoS Genet..

[67] Kowalczyk J., Palusinska M., Wroblewska-Swiniarska A., Pietras Z., Szewc L., Dolata J., Jarmolowski A., Swiezewski S. (2017). Alternative polyadenylation of the sense transcript controls antisense transcription of Arabidopsis seed dormancy QTL gene DOG1. Mol. Plant.

[68] Liu Y., Koornneef M., Soppe W.J.J. (2007). The absence of histone H2B monoubiquitination in the *Arabidopsis hub1 (rdo4)* mutant reveals a role for chromatin remodeling in seed dormancy. Plant Cell.

[69] Liu Y., Geyer R., van Zanten M., Carles A., Li Y., Hörold A., van Nocker S., Soppe W.J.J. (2011). Identification of the Arabidopsis REDUCED DORMANCY 2 gene uncovers a role for the polymerase-associated factor 1 complex in seed dormancy. PLoS ONE.

[70] Mortensen S.A., Sønderkær M., Lynggaard C., Grasser M., Nielsen K.L., Grasser K.D. (2011). Reduced expression of the DOG1 gene in Arabidopsis mutant seeds lacking the transcript elongation factor TFIIS. FEBS Lett..

[71] Mortensen S.A., Grasser K.D. (2014). The seed dormancy defect of Arabidopsis mutants lacking the transcript elongation factor TFIIS is caused by reduced expression of the DOG1 gene. FEBS Lett..

[72] Jackson J.P., Johnson L., Jasencakova Z., Zhang X., Perez-Burgos L., Singh P.B., Cheng X., Schubert I., Jenuwein T., Jacobsen S.E. (2004). Dimethylation of histone H3 lysine 9 is a critical mark for DNA methylation and gene silencing in *Arabidopsis thaliana*. Chromosoma.

[73] Zheng J., Chen F., Wang Z., Cao H., Li X., Deng X., Soppe W.J.J., Li Y., Liu Y. (2012). A novel role for histone methyltransferase KYP/SUVH4 in the control of Arabidopsis primary seed dormancy. New Phytol..

[74] Dekkers B.J., Pearce S.P., van Bolderen-Veldkamp R.P.M., Holdsworth M.J., Bentsink L. (2016). Dormant, after-ripened *Arabidopsis thaliana* seeds are distinguished by early transcriptional differences in the imbibed state. Front. Plant Sci..

[75] Iglesias-Fernández R., Rodríguez-Gacio M.C., Matilla A.J. (2011). Progress in research on dry afterripening. Seed Sci. Res..

[76] Chahtane H., Kim W., Lopez-Molina L. (2017). Primary seed dormancy: A temporally multilayered riddle waiting to be unlocked. J. Exp. Bot..

[77] Morscher F., Kranner I., Arc E., Bailly C., Roach T. (2015). Glutathione redox state, tocochromanols, fatty acids, antioxidant enzymes and protein carbonylation in sunflower seed embryos associated with after-ripening and ageing. Ann. Bot..

[78] Carrera E., Holman T., Medhurst A., Dietrich D., Footitt S., Theodoulou F.L., Holdsworth M.J. (2008). Seed after-ripening is a discrete developmental pathway associated with specific gene networks in Arabidopsis. Plant J..

[79] Vats S., Kumawat S., Kumar V., Patil G.B., Joshi T., Sonah H., Sharma T.R., Deshmukh R. (2019). Genome editing in plants: Exploration of technological advancements and challenges. Cells.

[80] Corte L.E.D., Mhmaoud L.M., Moraes T.S., Mou Z., Grosser J.W., Dutt M. (2019). Development of improved fruit, vegetable, and ornamental crops using the CRISPR/Cas9 genome editing technique. Plants.

