# Supplementary material for: Delay of Germination-1 (DOG1): A Key to Understanding Seed Dormancy
Source: Plants (Basel). 2020 Apr 9;9(4):480. doi: 10.3390/plants9040480 (PMC7238029; doi:10.3390/plants9040480)
Supplement: Supplementary file 1 [file plants-09-00480-s001.zip › supplementary/Additional file 1.pdf]

Table S1: Genes and proteins included in the phylogenetic tree (Fig. 1)

Plant species	Gene or Protein	Accession Number
Arabidopsis thaliana	AtDOG1	NP_001330541
Arabidopsis thaliana	AtDOGL1	NM_117982
Arabidopsis thaliana	AtDOGL2	NM_117984
Arabidopsis thaliana	AtDOGL3	NM_117985
Arabidopsis thaliana	AtDOGL4	NM_117981
Arabidopsis thaliana	AtDOGL5	NP_188106
Brassica rapa	BrDOG1	RID45165
Lepidum sativum	LesaDOG1	ACV41800
Lactuca sativa	LsDOG1	ALS87234
Sisymbrium officinale	SoDOG1	AIN76390
Brachypodium distachyon	BdDOG1L1	XP_003565204
Brachypodium distachyon	BdDOG1L2	XP_003565926
Brachypodium distachyon	BdDOG1L3	XP_003567594
Brachypodium distachyon	BdDOG1L4	XP_003560850
Brachypodium distachyon	BdDOG1L5-1	XP_003566099
Brachypodium distachyon	BdDOG1L5-3	XP_003564437
Brachypodium distachyon	BdDOG1L5-4	XP_003564436
Hordeum vulgare	HvDOG1L1	BAJ05340
Hordeum vulgare	HvDOG1L2	AK248238
Hordeum vulgare	HvDOG1L3	BE601551
Hordeum vulgare	HvDOG1L5-2	BAJ94197
Hordeum vulgare	HvDOG1L5-3	BAJ90602
Oryza sativa	OsDOG1L1	AK106273
Oryza sativa	OsDOG1L3	BAB64034
Oryza sativa	OsDOG1L4	CM000126
Oryza sativa	OsDOG1L5-1	NP_001049945
Oryza sativa	OsDOG1L5-3	EAZ36566
Oryza sativa	OsDOG1L5-4	EAY98526
Sorghum bicolor	SbDOG1L1	XP_002455164
Sorghum bicolor	SbDOG1L2	XP_002441508
Sorghum bicolor	SbDOG1L4	XP_002456383
Sorghum bicolor	SbDOG1L5-1	XP_002457410
Triticum aestivum	TaDOG1L1	BAJ05339
Triticum aestivum	TaDOG1L2	AK332921
Triticum aestivum	TaDOG1L4	AK330689

Triticum aestivum	TaDOG1L5-1	AK330559
Zea mays	ZmDOG1L2	NP_001152723
Zea mays	ZmDOG1L3	NP_001145406
Zea mays	ZmDOG1L4	ACG36725
Zea mays	ZmDOG1L5-1	NP_001132337
Zea mays	ZmDOG1L5-3	ACN26043
Zea mays	ZmDOG1L5-4	NP_001169220
