# Supplementary material for: Delay of Germination-1 (DOG1): A Key to Understanding Seed Dormancy
Source: Plants (Basel). 2020 Apr 9;9(4):480. doi: 10.3390/plants9040480 (PMC7238029; doi:10.3390/plants9040480)
Supplement: Supplementary file 1 [file plants-09-00480-s001.zip › supplementary/Additional file 2-1.pdf]

```
AtDOG1         1 ------------------------------------------------------------
AtDOGL1        1 ------------------------------------------------------------
AtDOGL2        1 ------------------------------------------------------------
AtDOGL3        1 ------------------------------------------------------------
AtDOGL4        1 ------------------------------------------------------------
AtDOGL5        1 ------------------------------------------------------------
BdDOG1L1       1 ------------------------------------------------------------
BdDOG1L2       1 ------------------------------------------------------------
BdDOG1L3       1 ------------------------------------------------------------
BdDOG1L4       1 ------------------------------------------------------------
BdDOG1L5-1     1 ------------------------------------------------------------
BdDOG1L5-3     1 ---------------------------------------------------MESRRGGG-
BdDOG1L5-4     1 ---------------------------------------------------MESRRGGG-
BrDOG1         1 ------------------------------------------------------------
HvDOG1L1       1 ------------------------------------------------------------
HvDOG1L2       1 ------------------------------------------------------------
HvDOG1L3       1 ------------------------------------------------------------
HvDOG1L5-2     1 ---------------------------------------------------MESRRGGGG
HvDOG1L5-3     1 ------------------------------------------------------------
LesaDOG1       1 ------------------------------------------------------------
LsDOG1         1 ------------------------------------------------------------
OsDOG1L1       1 ------------------------------------------------------------
OsDOG1L3       1 ------------------------------------------------------------
OsDOG1L4       1 ------------------------------------------------------------
OsDOG1L5-1     1 ------------------------------------------------------------
OsDOG1L5-3     1 ------------------------MAPSSLLIIFF---------------LLPTSRSSAL
OsDOG1L5-4     1 ------------------------------------------------------------
SbDOG1L1       1 ------------------------------------------------------------
SbDOG1L2       1 ------------------------------------------------------------
SbDOG1L4       1 ------------------------------------------------------------
SbDOG1L5       1 ------------------------------------------------------------
SoDOG1         1 ------------------------------------------------------------
TaDOG1L1       1 ------------------------------------------------------------
TaDOG1L2       1 ------------------------------------------------------------
TaDOG1L4       1 ------------------------------------------------------------
TaDOG1L5-1     1 ------------------------------------------------------------
ZmDOG1L2       1 ------------------------------------------------------------
ZmDOG1L3       1 ------------------------------------------------------------
ZmDOG1L4       1 ------------------------------------------------------------
ZmDOG1L5-1     1 ------------------------------------------------------------
ZmDOG1L5-3     1 --------------------------------------------MELYSGYLDDHFNPHK
ZmDOG1L5-4     1 MGDTSSSSHSRQDPSLLGYGFHGAIANSTPPANFFDQGGGGTYFGELEEAFMHQVASLRR
```

```
AtDOG1        1 ------------------------------------------------------------
AtDOGL1       1 ------------------------------------------------------------
AtDOGL2       1 ------------------------------------------------------------
AtDOGL3       1 ------------------------------------------------------------
AtDOGL4       1 ------------------------------------------------------------
AtDOGL5       1 ------------------------------------------------------------
BdDOG1L1      1 ------------------------------------------------------------
BdDOG1L2      1 ------------------------------------------------------------
BdDOG1L3      1 ------------------------------------------------------------
BdDOG1L4      1 ------------------------------------------------------------
BdDOG1L5-1    1 MDYAS-----PGG------TDTSTDPGIAKKNQ---------------------------
BdDOG1L5-3    9 --PAAAAAEDARGPMPGFGAPQHTIPTDVNIMQTSRVTDFGALAQSAGFRIEDLANLSAN
BdDOG1L5-4    9 --PAAAAAEDARGPMPGFGAPQHTIPTDVNIMQTSRVTDFGALAQSAGFRIEDLANLSAN
BrDOG1        1 ------------------------------------------------------------
HvDOG1L1      1 ------------------------------------------------------------
HvDOG1L2      1 ------------------------------------------------------------
HvDOG1L3      1 ------------------------------------------------------------
HvDOG1L5-2   10 PAAAAAAAGDPRGPMPGFGAPQHTIPTDVNIMQPSRVADFGALAHSAGFRIEDLANFSTN
HvDOG1L5-3    1 -----MAEASPR-------TETS-DDTDENLM----------------------------
LesaDOG1      1 ------------------------------------------------------------
LsDOG1        1 ------------------------------------------------------------
OsDOG1L1      1 ------------------------------------------------------------
OsDOG1L3      1 ------------------------------------------------------------
OsDOG1L4      1 ------------------------------------------------------------
OsDOG1L5-1    1 -----MADASSR------TDTSTVLDTDDKN-----------------------------
OsDOG1L5-3   22 VTHATVSSTVAFQELLLCPNPSSKSSSNLTAGGHLPPLAVAAAAAAAQQHGVAVVGMAAH
OsDOG1L5-4    1 MFVASSPRKCPQGPCQAARTANQLQPGSSGSHR---------------------------
SbDOG1L1      1 ------------------------------------------------------------
SbDOG1L2      1 ------------------------------------------------------------
SbDOG1L4      1 ------------------------------------------------------------
SbDOG1L5      1 -----MADASPR-------TETSTDDTDDNHG----------------------------
SoDOG1        1 ------------------------------------------------------------
TaDOG1L1      1 ------------------------------------------------------------
TaDOG1L2      1 ------------------------------------------------------------
TaDOG1L4      1 ------------------------------------------------------------
TaDOG1L5-1    1 -----MADASSR------TDNSTVLDNDGKIY----------------------------
ZmDOG1L2      1 ------------------------------------------------------------
ZmDOG1L3      1 ------------------------------------------------------------
ZmDOG1L4      1 ------------------------------------------------------------
ZmDOG1L5-1    1 -----MADASPR-------TETSTDDTDDNNG----------------------------
ZmDOG1L5-3   17 LSIAGSAVSPPEYMASASPAQFAAAPLRMGYGRPAPAPPPPVMGMWSSEPFRVDSGSAHA
ZmDOG1L5-4   61 TQQAATVSAPHHGDTKPFPTAAGATAATATATARPPPTLDIFPAWPMRSRLHTPKECSNV
```

```
AtDOG1          1 ------------------------------------------------------------
AtDOGL1         1 ------------------------------------------------------------
AtDOGL2         1 ------------------------------------------------------------
AtDOGL3         1 ------------------------------------------------------------
AtDOGL4         1 ------------------------------------------------------------
AtDOGL5         1 ------------------------------------------------------------
BdDOG1L1        1 ------------------------------------------------------------
BdDOG1L2        1 ------------------------------------------------------------
BdDOG1L3        1 ------------------------------------------------------------
BdDOG1L4        1 ------------------------------------------------------------
BdDOG1L5-1     23 ------------------------------------------------------------
BdDOG1L5-3     67 TLFNLKPNNHTFISDPLQFGNYGK------------------------------------
BdDOG1L5-4     67 TLFNLKPNNHTFISDPLQFGNYGKSISPTDLATTAAAAAAAAITTVDPQALLQQKGVQSN
BrDOG1          1 ------------------------------------------------------------
HvDOG1L1        1 ------------------------------------------------------------
HvDOG1L2        1 ------------------------------------------------------------
HvDOG1L3        1 ------------------------------------------------------------
HvDOG1L5-2     70 NLFNLKPNTHAYTSDPLQFGNYGKSISPTDLATT----AAAAVTAVDPQALLQQKAVQPN
HvDOG1L5-3     20 ------------------------------------------------------------
LesaDOG1        1 ------------------------------------------------------------
LsDOG1          1 ------------------------------------------------------------
OsDOG1L1        1 ------------------------------------------------------------
OsDOG1L3        1 ------------------------------------------------------------
OsDOG1L4        1 ------------------------------------------------------------
OsDOG1L5-1     21 ------------------------------------------------------------
OsDOG1L5-3     82 QGMAAATAADRFCLPRMAAAAAAAASQVEN------------------------------
OsDOG1L5-4     34 ------------------------------------------------------------
SbDOG1L1        1 ------------------------------------------------------------
SbDOG1L2        1 ------------------------------------------------------------
SbDOG1L4        1 ------------------------------------------------------------
SbDOG1L5       21 ------------------------------------------------------------
SoDOG1          1 ------------------------------------------------------------
TaDOG1L1        1 ------------------------------------------------------------
TaDOG1L2        1 ------------------------------------------------------------
TaDOG1L4        1 ------------------------------------------------------------
TaDOG1L5-1     22 ------------------------------------------------------------
ZmDOG1L2        1 ------------------------------------------------------------
ZmDOG1L3        1 ------------------------------------------------------------
ZmDOG1L4        1 ------------------------------------------------------------
ZmDOG1L5-1     21 ------------------------------------------------------------
ZmDOG1L5-3     77 TSASTVN-----------------------------------------------------
ZmDOG1L5-4    121 TADSTDDSESSSKNHSNHSSDQLGAAAAAN------------------------------
```

```
AtDOG1         1 ------------------------------------------------------------
AtDOGL1        1 ------------------------------------------------------------
AtDOGL2        1 ------------------------------------------------------------
AtDOGL3        1 ------------------------------------------------------------
AtDOGL4        1 ------------------------------------------------------------
AtDOGL5        1 ------------------------------------------------------------
BdDOG1L1       1 ------------------------------------------------------------
BdDOG1L2       1 ------------------------------------------------------------
BdDOG1L3       1 ------------------------------------------------------------
BdDOG1L4       1 ------------------------------------------------------------
BdDOG1L5-1    23 ---------------------------------------MFEQGHVA-ALKAFNSGDKS-
BdDOG1L5-3    91 ----------------------------------------FEQGHLA-APTGSDSSDKS-
BdDOG1L5-4   127 IVALRTRNSENWGESSMADTSPRTDTSTDPDIDVDERNQMFEQGHLA-APTGSDSSDKS-
BrDOG1         1 ------------------------------------------------------------
HvDOG1L1       1 ------------------------------------------------------------
HvDOG1L2       1 ------------------------------------------------------------
HvDOG1L3       1 ------------------------------------------------------------
HvDOG1L5-2   126 LVALRTRNNENWGESSMADTSPRTDTSTDPDIDIDERNQMFEQGQLA-APTASDSSDKS-
HvDOG1L5-3    20 ----------------------------------------LEPGNAA-LAVASDSSDRS-
LesaDOG1       1 ------------------------------------------------------------
LsDOG1         1 ------------------------------------------------------------
OsDOG1L1       1 ------------------------------------------------------------
OsDOG1L3       1 ------------------------------------------------------------
OsDOG1L4       1 ------------------------------------------------------------
OsDOG1L5-1    21 ---------------------------------------QMVDGQSG-AIVPSNSSDRSD
OsDOG1L5-3   111 -----WGDSGVIVSSPFTDDTSTDLDDSADKHHLHALVGGGDGGDDAGEQRGADSSAVS-
OsDOG1L5-4    34 ---------------------------------------IDSMLGFA-AESARRIRSAR-
SbDOG1L1       1 ------------------------------------------------------------
SbDOG1L2       1 ------------------------------------------------------------
SbDOG1L4       1 ------------------------------------------------------------
SbDOG1L5      21 ----------------------------------------LEPGPGA-LGVASDSSDRS-
SoDOG1         1 ------------------------------------------------------------
TaDOG1L1       1 ------------------------------------------------------------
TaDOG1L2       1 ------------------------------------------------------------
TaDOG1L4       1 ------------------------------------------------------------
TaDOG1L5-1    22 ---------------------------------------RLEQGQSGGAIMASNSSDRSD
ZmDOG1L2       1 ------------------------------------------------------------
ZmDOG1L3       1 ------------------------------------------------------------
ZmDOG1L4       1 ------------------------------------------------------------
ZmDOG1L5-1    21 ----------------------------------------LEPGRGG-L-VASDSSDRS-
ZmDOG1L5-3    84 -------------------------------------TKLEETRLEDDAQVALEPARSTE
ZmDOG1L5-4   151 ------------------------------------MASQFEQASQQQLQHKNMATSSTP
```

```
AtDOG1        1 ------------------------------------------------------------
AtDOGL1       1 ------------------------------------------------------------
AtDOGL2       1 ------------------------------------------------------------
AtDOGL3       1 ------------------------------------------------------------
AtDOGL4       1 ------------------------------------------------------------
AtDOGL5       1 ------------------------------------------------------------
BdDOG1L1      1 ------------------------------------------------------------
BdDOG1L2      1 ------------------------------------------------------------
BdDOG1L3      1 -------------------------------MPTSLQRDAAAAAAVSKERRAVRQP----
BdDOG1L4      1 ------------------------------------------------------------
BdDOG1L5-1   42 --KAKLGQNTLRRLAQNR---------DAARKSRLRK---KAYVQKLESSSLKLAQLEQE
BdDOG1L5-3  109 -RDQ-LDHKSLRRLAQNR---------EAARKSRLRK---KAYIQNLETSRLKLTQLEQE
BdDOG1L5-4  185 -RDQ-LDHKSLRRLAQNR---------EAARKSRLRK---KAYIQNLETSRLKLTQLEQE
BrDOG1        1 ------------------------------------------------------------
HvDOG1L1      1 ------------------------------------------------------------
HvDOG1L2      1 ------------------------------------------------------------
HvDOG1L3      1 ------------------------------------------------------------
HvDOG1L5-2  184 -RDK-LDHKSLRRLAQNR---------EAARKSRLRK---KAYIQNLESSRLKLTQLEQE
HvDOG1L5-3   38 -RDRNGDQKTMRRLAQNR---------EAARKSRLRK---KAYVQQLENSRLKLTQLEQE
LesaDOG1      1 ------------------------------------------------------------
LsDOG1        1 ------------------------------------------------------------
OsDOG1L1      1 ------------------------------------------------------------
OsDOG1L3      1 -------------------------------MRTPAAPCARACPPHAKKPRPIYAGPGWE
OsDOG1L4      1 ------------------------------------------------------------
OsDOG1L5-1   41 RSDKPMDQKVLRRLAQNR---------EAARKSRLRK---KAYVQQLESSKLKLASLEQE
OsDOG1L5-3  165 -KERRGDQKMQRRLAQNR---------EAARKSRMRK---KAYIQQLESSRSKLMHLEQE
OsDOG1L5-4   53 --HTNFSRLLISAQAQEFNMVYASPGTDASTDPDIDKNIRMAYVQQLEDSRMKLTQLEQE
SbDOG1L1      1 ------------------------------------------------------------
SbDOG1L2      1 ------------------------------------------------------------
SbDOG1L4      1 ------------------------------------------------------------
SbDOG1L5     39 -KDKHGDQKTLRRLAQNR---------EAARKSRLRK---KAYVQQLENSRLKLTQLEQE
SoDOG1        1 ------------------------------------------------------------
TaDOG1L1      1 ------------------------------------------------------------
TaDOG1L2      1 ------------------------------------------------------------
TaDOG1L4      1 ------------------------------------------------------------
TaDOG1L5-1   43 RSDKPLDQKTLRRLAQNR---------EAARKSRLRK---KSYVQQLESSKLKLAQLEQE
ZmDOG1L2      1 ------------------------------------------------------------
ZmDOG1L3      1 ------------------------------------------------------------
ZmDOG1L4      1 ------------------------------------------------------------
ZmDOG1L5-1   38 -KDKHGDQKTLRRLAQNR---------EAARKSRLRK---KAYVQQLENSRLKLTQLEQE
ZmDOG1L5-3  107 QETSRPPERAQRRLAQNR---------EAARKSRLRK---KAYIQQLETSRMKLSQLELE
ZmDOG1L5-4  175 RTGKALDPKVIRRLAQNR---------EAARKSRLRK---KAYIQQLESCKLKLSQMEQD
```

```
AtDOG1         1 -----------------MGSSSKNIEQ--------AQDS-YLEWMSLQSQRIPELKQLLA
AtDOGL1        1 ------------------------------------------------------------
AtDOGL2        1 --------------------------------------------MSLQTKHIDDLKEALM
AtDOGL3        1 ---------------MATSSSSYGIEQ--------LQKGCYYEWMSVQAKHIVDLKEALM
AtDOGL4        1 ----------------MSKMRNLVEEK---------FLEFYESWVIQLELYLHQLLIAHN
AtDOGL5        1 -------------------MSQETAIA--------SFKKFQQSWIEQLRNHLNHLRSAQN
BdDOG1L1       1 -MTAAS----RPQS-NG--EPLVDG-E--------PFTKFFGCWISEQSRDLAALREAAA
BdDOG1L2       1 -----------------MSAGMEAELE--------AAQRRFRLWFRGLRSLRRDLRSARW
BdDOG1L3      26 -----------------ARIMAHVA----------DMAAFYDAWVGREEEIVADLTAALS
BdDOG1L4       1 -----------------------MDMT--------RYFACHQQWIAGQEAGLGELTAAAA
BdDOG1L5-1    88 LLRARQQGYVTST--LGEQPHPANGNG------ALALDVEYGRWLEEHNKQIDELRAAIS
BdDOG1L5-3   155 LQRARQQGIFISS--SGDQSHSTSGNG------ALAFDMEYARWLEEHNKHINELRAGVN
BdDOG1L5-4   231 LQRARQQGIFISS--SGDQSHSTSGNG------ALAFDMEYARWLEEHNKHINELRAGVN
BrDOG1         1 -----------------MENKGKNIEQ--------AQASCYQQWMSIQSQRVPELKQALA
HvDOG1L1       1 -MTATS----RPQHHNGSLARASDGGE--------SFAKFFECWILEQSRDLAALR-AAA
HvDOG1L2       1 -----------------------MELE--------AARRRFQLWLRGLRSLRRDLRTARW
HvDOG1L3       1 --------------------MAHAG----------DMTAFYGAWVGREEEIVSDLTAALG
HvDOG1L5-2   230 LQRARQQGIFISS--SGDQAHSAGGNG------AVAFDMEYARWLEEHNKHINELRAAAN
HvDOG1L5-3    85 LQRARQQGIFISS--SADQSHSMSGNG------ALAFDTEYARWLEEHNRQVNELRAAVN
LesaDOG1       1 -----------------MGSSKKNIEE--------AQESCYQEWMNLQSQRVPDLKHLLA
LsDOG1         1 -------------------MAKQMKHQ--------QFQCCYKNWVAQQQLDLDELLQTLT
OsDOG1L1       1 -MPPPSP---HPPHRNGNHVPAPSG-E--------SFAKFFECWISEQSRDLAALRSAAS
OsDOG1L3      30 GQRMPASYLQPRRGTNGRRIMEHGAGE--------EMVAFYEAWVGREERIVADLTDAL-
OsDOG1L4       1 -----------------------MGAA--------RHVACYQRWIAGQEAGLGELEASSA
OsDOG1L5-1    89 INKARQQGIYISS--SGDQTHAMSGNG------AMTFDLEYARWLEEQNKQINELRTAVN
OsDOG1L5-3   212 LQRARQQGIFIATGGSGDHGHSIGGNGG-----TLAFDLEYARWLDEHQRHINDLRVALN
OsDOG1L5-4   111 LQRARQQGIIIST--SGDQQRSTSENE------ALAFNMEYMRWLEEHNKQINELRSAVH
SbDOG1L1       1 -MPATFPPPPPPPPPSHNLPPLLSSNE--------SFSKFFESWIGEQSRDLEELRAAAS
SbDOG1L2       1 ---------------------MELELE--------AATRRFHLWFRGLRSLRRDLASARW
SbDOG1L4       1 -----------------------MDMA--------RYESCYRHWIAGQEAGLAELEAASA
SbDOG1L5      86 LQRARQQGIFISS--SVDQSHSMSGNG------ALAFDMEYARWLEEHNRQISELRAGVS
SoDOG1         1 -----------------MEDKVKNIEQ--------AQESCYVKWMSLQSQRIPELKQALA
TaDOG1L1       1 -MTATS----RPQHPNGSLAPASDGGE--------SFAKFFECWILEQSRDLAALR-AAA
TaDOG1L2       1 -----------------------MELE--------AATRRFQLWLRGLRSLRRDLRTARW
TaDOG1L4       1 -----------------------MAMA--------RYVAFHQQWIAGQQAGLGELAEAAA
TaDOG1L5-1    91 LQKARQQGIFISS--SGDQTHAMSGNG------AMTFDLEYTRWLEDQNKQINELRTAVN
ZmDOG1L2       1 ---------------------MELELE--------AAARRFHLWFRGLRSLRRDLASARW
ZmDOG1L3       1 ------------------------------------MAAFYDAWVGREEQIVAELTAALA
ZmDOG1L4       1 -----------------------MDMA--------RYESCYRHWIAGQEAGLAELEAASA
ZmDOG1L5-1    85 LQRARQQGIFISS--SVDQSHSMSGNG------ALAFDMEYARWLEEHNRQISELRAGVS
ZmDOG1L5-3   155 LQRARQQGAYANS---GSMGDSALGYRCPIDPGVSVFEIDYSHWVDEQKRHTAELTSALQ
ZmDOG1L5-4   223 MQRARTQGLFLGG-----DPGASTSSG------AAMFDVEYARWLDNHSRRLAELNGALH
```

```
AtDOG1        35 QRRSHG----DEDNDNKLRKLTGKIIGDFKNYAAKRADLAHRCSSNYYAPTWNSPLEN-A
AtDOGL1        1 ------------------------------------------------------------
AtDOGL2       17 CQ--------RNNDD-KLEDLVGKIVNDYHTYAGKRSELSYRCCAHYFAPSWNTPIEN-S
AtDOGL3       38 SH--------RSKEDHKLEELVGKIVNDFQKYTEKRSELSRRSCSSYFAPSWNSPLEN-G
AtDOGL4       36 NN---------TMSETELRHLISKLTTHHKAYYTAKWAAIREDVLAFFGSVWLNPLEN-A
AtDOGL5       34 HHRN-S----ATGDEERLREAVDRVMEHFREYHRAKWAATDKDVIEVMASPWASALER-S
BdDOG1L1      44 AAS---------SSSADLRRLVDRVLGHYEHYYRAKSAAAAADVRAMFAPSWISTTES-L
BdDOG1L2      36 AG----------DDPAQLGKLAGGFVAHFSDYCAAR---AELDPVLLLSAPWASPAERGA
BdDOG1L3      59 LSLS-------ARRREALAPLVDAAMDHVATYYEHKARLADRDVVAALDPRWLNPLER-T
BdDOG1L4      30 NAAARR------ATEAELRAVVERCLRGYEEYATTRRAMAPEDGAAFFAPPWCTTFEK-A
BdDOG1L5-1   140 ----------ARATDGDLHAIVENIMAHVDEIFRLKSVATKANAFHVLAGAWTTPVER-C
BdDOG1L5-3   207 ----------AHAGDDDLRSIVDCIMAHYDEFFRLKGVAAKADVFHVLSGMWKTPAER-C
BdDOG1L5-4   283 ----------AHAGDDDLRSIVDCIMAHYDEFFRLKGVAAKADVFHVLSGMWKTPAER-C
BrDOG1        36 QRRTHEGTADAAADDNKLRELTQNIIGDFKNYAGKRADLSHRCSSSYYAPSWNTPLEN-A
HvDOG1L1      47 TAR---------PHDADLRRLVDRVLGHYENYYRAKSAAASADVLPMFAPSWISATES-L
HvDOG1L2      30 A-----------DDPAQLAKLVAGYVSHFADYCAAR---AELDPVWTLAAPWASPVERGA
HvDOG1L3      31 -----------ARRRDALAPLVDAAMDHVATLYEHKASLADRDVVAALDQRWLNPLER-T
HvDOG1L5-2   282 ----------AHAGDDDLRSIVDSIMAQYDEFFRLKGVAAKADVFHVLSGMWKTPAER-C
HvDOG1L5-3   137 ----------AHAGDTELRSVVEKIMSHYDEIFKQKGNAAKADVFHVLSGMWKTPAER-C
LesaDOG1      36 QRRSNK---ANTDNDNKLRELLEKIIGDFKKYAGKRADLSHRCSSNYYAPSWNTPLEN-A
LsDOG1        34 NYP---------TDVDYLQLITKKIVSHFENYHNSRAELAKHDGPSFLAPSWGSTFEN-S
OsDOG1L1      48 AATNPA----APPDDAELHRLVNRVLGHYEHYYRTKSAAASTDVLRMFSPSWTSTTEN-L
OsDOG1L3      81 -LPA-------RRRRDVLAPLVDAAVGHVSEYYERKARLADRDVVAALDPRWLNPLER-T
OsDOG1L4      30 NAAAGR------ATDGELRAVVERCMRGYAEYVSTRRALAREDGAALFAPPWCTSFEN-S
OsDOG1L5-1   141 ----------AHASDSDLRLIVDGIMAHYDEIFRLKGVAAKADVFHILSGMWKTPAER-C
OsDOG1L5-3   267 ----------AQMSDDELCELVDAVMMHYDQVFRLKSFATKSDVFHVLSGMWMSPAER-F
OsDOG1L5-4   163 ----------THAGDDDLQSIVSNFMAHHEEIFRIKGLAAKADALHVLSATWRTPLER-C
SbDOG1L1      52 AEP--------AAPEADLRRLVDQVMGHYAQYYSTKAAAAAGDVSMMFTPSWTSTTEN-L
SbDOG1L2      32 SD----------DAAAQLPALVGRFVAHLESYCAAR---AGLDPVWTLSAPWATPAERGA
SbDOG1L4      30 NAAAGR------ATDAELRAVVERCMLGYQDYATRRRALSREDGAAFFAPPWCTAFEN-S
SbDOG1L5     138 ----------AHASDTDLRSVVDKIMSHYDEIFRLKGNAAKADVFHVLSGMWKTPAER-C
SoDOG1        36 QRRSHEGTAAASADDNKLRDLIQKIIGDFKDYAGKRSDLSTRCSSSYYAPSWNTPLEN-A
TaDOG1L1      47 TAR---------PDDADLRRLVDRVLGHYEHYYRAKSAAASADVLPMFAPSWISATES-L
TaDOG1L2      30 A-----------DDPAQLAKLVAGYVSHFADYCAAR---AELDPVWTLAAPWASPVERGA
TaDOG1L4      30 NAAAGR------ATDAELKTVVERCMRGYQEYAASRRAMARENGAAFVAPPWCTAFEN-S
TaDOG1L5-1   143 ----------AHASDSDLRLIVDGIMGHYDEIFKVKGVAAKADVFHILSGMWKTPAER-C
ZmDOG1L2      32 SD----------DPTAQLPAQVGRFVAHLESYCAAR---AGLDPVWTLSAPWATPAERGA
ZmDOG1L3      25 -LQP-------RRRGDALALLVDGAVAHVAAYYEHKSRLADRDVVAALDPRWLNPIER-T
ZmDOG1L4      30 NAAAGR------ATDAELRTVVERCMLGYQDYATRRRALSREDGVAFFAPPWCTAFEN-S
ZmDOG1L5-1   137 ----------AHASDTDLRSVVDKIMSHYDEIFRLKGNAAKADVFHVLSGMWKTPAER-C
ZmDOG1L5-3   212 G---------QQTSELELRLLVETGLSNYEHLFKIKAAAANADVFYVMSGLWKTPAER-F
ZmDOG1L5-4   272 ----------AHLADGDLRAIVDDALTHHDELFQLKAMAAKSDVFHLITGVWTTPAER-C
```

```
AtDOG1        90  LIWMGGCRPSSFFRLVYALCGSQTEIRVTQFLRNIDGYESSGGGGG--ASLSDLSAEQL-
AtDOGL1        1  -MALGETR--------------------------------GGIGGG--ESMSDLTAEQL-
AtDOGL2       67  MLWMGGCRPSSFIRLIYALCGSQAETQLSQYLLKIDDDFDINHGG----FMSDLTATQL-
AtDOGL3       89  LLWMGGCRPSSFIRVIYSLCGSQAETQLSQYLLKIDENVEVNHGG----SMSDLNASQL-
AtDOGL4       86  CSWLTGWKPSMVFRMVDRLRKSRVVLVEAQVK----------------------------
AtDOGL5       88  LQWVGGWRPTTLFHLVYTESSILFESRIVDILRG---FRTGD--------LSDLSPSQFR
BdDOG1L1      94  YLWCGGWRPTAALHLLYSKSGAQLEAQLPAFLDGTGSLRGDD--------LGGLSADQL-
BdDOG1L2      83  AYWLAGWRPTTVVHLLYTESSRRFEAQLPDLLLG---VRSGN--------LGDLSPAQL-
BdDOG1L3     111  FLWAWGWKPALVFRFVDEAAVGS---AQQR------------------------------
BdDOG1L4      83  VLWLGGCRPSLSIRLLYCVSSEGLEAQLQEFLSGHGRAGGDDMIRPTGSGLLGINAMQL-
BdDOG1L5-1   189  FLWLSGFRPSELPKLLASQLEPLTEKQLAS------------------------------
BdDOG1L5-3   256  FMWLGGFRSSELLKLLAGQLEPLTEQQLAG------------------------------
BdDOG1L5-4   332  FMWLGGFRSSELLKLLAGQLEPLTEQQLAG------------------------------
BrDOG1        95  LIWMGGCRPSSFFRLVYALCGSQTEIRVTQFLRNIDGYDYSGSGG---ASLSDLTAEQL-
HvDOG1L1      97  YLWCGGWRPTAAVQLLYSKSGVQLEAQLPAFLDG-GSLGDGD--------LGGLSAEQL-
HvDOG1L2      76  AHWLAGWRPTTLVHLLYTESGRRFEAQLPDLLLG---VRSGN--------LGDLSPAQL-
HvDOG1L3      79  FLWAWGWRPALVFRFVDGSGIG----PRQR------------------------------
HvDOG1L5-2   331  FMWLGGFRSSELLKLLAGQLEPLTEQQLTG------------------------------
HvDOG1L5-3   186  FLWLGGFRPSELLKLLSTQLEPLTEQQLSG------------------------------
LesaDOG1      92  LLWMGGCRPSSFFRLVYALCGSETEIRVTQYLRDIDGLESSGGMG---TSLSDLTAEQL-
LsDOG1        84  FLWIGGCRPALMIRLVYALCGSHLNTHLEEFLEG------VRHGN-----IGEISSLQL-
OsDOG1L1     103  YLWCSGWRPTAALHLLYSKSGAQLETQLPVFLAG-GGLGAGD--------LGDLSAEQL-
OsDOG1L3     132  FLWAWGWKPALVFRFADGAVAGGSSHQQQR------------------------------
OsDOG1L4      83  VLWIGGCRPSLTIRLLYSLSGEGLEEHIEEFISGRGALGAARG-----MGLLGITARQL-
OsDOG1L5-1   190  FLWLGGFRSSELLKLLVNQLEPLTEQQLLG------------------------------
OsDOG1L5-3   316  FMWLGGFRSSELLKVLASHLEPLTDQQLMG------------------------------
OsDOG1L5-4   212  FLWLGGFRPSDLLKLLADQLEPLTEQQLAS------------------------------
SbDOG1L1     103  YLWCGGWRPTAAIQLLYTKCGMQLEHRLPVFLDG-GGLNKDD--------LSDLSVAQL-
SbDOG1L2      79  AYWLAGWRPTTLVHLLYTESGRRLEAQLPDLLLG---VRSGN--------LGDLTPAQL-
SbDOG1L4      83  LLWLGGCRPSLTVRLLYNISGEGLEAQVEEMLGGLTHGVIPTG-------ALGITSAQL-
SbDOG1L5     187  FLWLGGFRPSEVLKLLSTQLEPLTEQQLSG------------------------------
SoDOG1        95  LIWMGGCRPSSFFRLVYALCGSQTEIRVTQFLRNIDGYDSSGSGG---VSLSDITAEQL-
TaDOG1L1      97  YLWCGGWRPTAAIQLLYSKSGVQLEAKLPAFLDG-GSLGDGD--------LGGLSAEQL-
TaDOG1L2      76  AHWLAGWRPTTLVHLLYTESGRRFEAQLPDLLLG---VRSGN--------LGDLSPAQL-
TaDOG1L4      83  VLWLGGCRPSLAIRLLYSISGEGLEEDIEEFVSGRGRGLAEEM------GLIGITATQL-
TaDOG1L5-1   192  FLWLGGFRPSELLKLLVNHLEPLTEQQMLG------------------------------
ZmDOG1L2      79  AYWLAGWRPTTLVHLLYTESGRRLEAQLPDLLLG---VRSGN--------LGDLTPAQL-
ZmDOG1L3      76  FLWAWGWKPALMFRFVESVGVG--LRLEQR------------------------------
ZmDOG1L4      83  VLWLGGCRPSLTVRLLYNLSGEGLEAQVEELLGGLSNGVIPTG-------ALGITSAQL-
ZmDOG1L5-1   186  FLWLGGFRPSEVLKLLSTQLEPLTEQQLSG------------------------------
ZmDOG1L5-3   262  FLWIGGFRPSEVLKILSPQLEPLAEAQRML------------------------------
ZmDOG1L5-4   321  FLWMGGFRPSDLLKTLLPQLDPLTEQQVVG------------------------------
```

```
AtDOG1        147 ------AKINVLHVKIIDEEEKMTKKVSSLQEDAADIPIATVAYEM--------------
AtDOGL1        25 ------FKINELHLKTVEAENKLTKVSASLQEDTADTPIAVAAFYK--------------
AtDOGL2       122 ------GKLNDLHLEVIKKEDKITKTSANFQDDVADLPIA--------------------
AtDOGL3       144 ------AKINDLHIKVIEKEDKITKKSANLQENVADMPIAIAAYAT--------------
AtDOGL4       118 -------KLEELRVKTKFDEQKIEREMERYQVAMADRKMVELARLG--------------
AtDOGL5       137 VPFVKGRTVSELQCETVKEENAITEELSEWQDDASDLV----------------------
BdDOG1L1      145 ------HAADQLQRRTIGREREIEEAAAAAQESLATGKMVELAT----------------
BdDOG1L2      131 ------AQIDELQRRAVAEEDALSREMARLQEGHGVVG----------------------
BdDOG1L3      138 ------RGLEDLRASTAAAEREVEREVAAMQESLAGPRVLAALRRQ--------------
BdDOG1L4      142 ------EQINNLHGRTIHEEGILSERLASLQEKIADRPLLPIVRER----EQERARAAAL
BdDOG1L5-1    219 --------ICSLRQSSQQAEDTLSRDMEVLLQSAAEIVAS--GTSPTWY-----------
BdDOG1L5-3    286 --------ICNLQQSSQQAEDALSQGMEALQQSLAETLAS-GSLGPAGS-----------
BdDOG1L5-4    362 --------ICNLQQSSQQAEDALSQGMEALQQSLAETLAS-GSLGPAGS-----------
BrDOG1        151 ------AKINVLHVKIIDEEEKMTKKVSSLQEDAADIPISTVAYAE--------------
HvDOG1L1      147 ------QAADQLHRRTIRREREIEEAAASAQESLATTRMVELAGK---------------
HvDOG1L2      124 ------AQIDELQRRTVAQEDELSREMARVQEGDGLVG----------------------
HvDOG1L3      105 ------RELEDLRAATAAAEKEVDREVAAVQESLAGPRVLEALRQR--------------
HvDOG1L5-2    361 --------ICNLQQSSQQAEDALSQGMEALQQSLAETLAS-GSLGPAGS-----------
HvDOG1L5-3    216 --------ICNLQQSSQQAEDALSQGMEALQQSLAETLA--GSIGSSGSGS---------
LesaDOG1      148 ------AKINILHVKIIEDEEKMTKKVASLQEDAADIPIATVAYEE--------------
LsDOG1        132 ------KRIDELHAKTIKEEDKLSSYMATLQEKIAGEPLVLLASGC--------------
OsDOG1L1      153 ------QAADQLQRITVSKEREIENAAASAQVS--------------------------
OsDOG1L3      162 ------RALERVRAATAEAEREVDREVAVVQESLAGPRVLAALRR---------------
OsDOG1L4      137 ------ELVNDLHRRTLRDEDALSDRLATLQEDVADRPLLPIVRER----ATAAAAALGA
OsDOG1L5-1    220 --------LSNLQQSSQQAEDALSQGMEALQQSLADTLA--GSLGPS-GS----------
OsDOG1L5-3    346 --------ICNLQQSSQQAEDALSQGMEALQQTLGDTLVSAAATVVSGGGG---------
OsDOG1L5-4    242 --------ICNQQQSSQEAEETLSQGMEIIQDSLAKTVAS--QLGRAGS-----------
SbDOG1L1      153 ------RAADQLQHRTISREREIEEVAATAQEALTSKTMVELAGGG--------------
SbDOG1L2      127 ------AQVDDLQRRTVAEEDALAREMALVQEGHGVVAAPSA------------------
SbDOG1L4      135 ------LLINDLHSRTVHQENALTDRLATLQEDIADRPLLPIVRQRGELAAAAAR-QGGA
SbDOG1L5      217 --------IGNLQQSSQQAEDALSQGMEALQQSLAETLA--GSLSSSGS-----------
SoDOG1        151 ------AKINVLHVKIIDEEEKMTKKVSSLQEDAADIPISTVAYAE--------------
TaDOG1L1      147 ------QAADQLQRRTIRGEREIEEAAAGAQESLATTKMVELAGK---------------
TaDOG1L2      124 ------AQIDELQRRTVAQEDELSREMARVQEGHGAVG----------------------
TaDOG1L4      136 ------QQINDLHRCTLRDEGYLTERLASLQENIADRPLLPIVREH----AVAATALVGQ
TaDOG1L5-1    222 --------LTNLQQSSQQAEDALSQGMEALQQSLAETLA--GSLGSSAGS----------
ZmDOG1L2      127 ------AQVDDLQRRTVAEEDALAREMALVQEGHGVVVLPAAP-----------------
ZmDOG1L3      104 ------RALEELRGATAVAEREVDLQVAAVQESLAGPRVLAALRR---------------
ZmDOG1L4      135 ------VLINDLHSRTVHQENALSDRLATLQEDIADRPLLPIVRQR-ELAAAARLGAAAA
ZmDOG1L5-1    216 --------ISNLQQSSQQAEDALSQGMEALQQSLAETLA--GSLSSSGS-----------
ZmDOG1L5-3    292 --------VGGLQHTSAQAEDALSQGMEKLQQNLAETLTA--EADPFGP-----------
ZmDOG1L5-4    351 --------ICSLQQSSQQAEEALSQGLEQLHQSLADTMAG-GSLTDDAN-----------
```

```
AtDOG1       187 ---------------ENVGE--PNVVVDQALDKQEEAMARLLVEADNLRVDTLAKIL-GI
AtDOGL1       65 ---------------EVIGQ--ADVVVERALDKHEEDMGGLLVEADKLRMTTLTKIV-DI
AtDOGL2      156 ---------------DVVH---ADVAVEDALDKHEEGMAVLLAEADKLRFETLRKIV-DV
AtDOGL3      184 ---------------DLMN---GDVVVEDALDKYEEGMAVLMVEADKLRFETLRKIV-DV
AtDOGL4      157 ----------------CHVGGESVMVVEAAVRGLSMGLEKMVKAADCVRLKTLKGIL-DI
AtDOGL5      175 ------------------------MGTSSDPDQRIRRLAEIVHRTDDLRLRTITRVV-EV
BdDOG1L1     183 ------------------GAMEAAG-LEREMEAKAEGMRRVLEMADGLRLDTMRAVV-AL
BdDOG1L2     163 -----------------------GDGD-LDVEGIVRRAGAVVAGADALRLRTLKRAV-EI
BdDOG1L3     178 -----------------LHSPRNG-EADEAVAAVGRSLRVLLAAGDALRERTVRGVV-GL
BdDOG1L4     192 PRGSASSNGLVGRFAAMGLAG-VDAEVDAAMESYTAGLAKLLEEADQLRLSTTRELATEI
BdDOG1L5-1   258 -----------------PAGSSGDTGQMSAAIGKLGAVESLLQQADELRLRILRDVQ-RI
BdDOG1L5-3   326 -----------------SSNVANYMGQMAMAMGKLGTLENFLRQADNLRLQTLQQMQ-RI
BdDOG1L5-4   402 -----------------SSNVANYMGQMAMAMGKLGTLENFLRQADNLRLQTLQQMQ-RI
BrDOG1       191 ---------------EHVGE--PNAMVDQALDKQEEAMATLLAEADNLRVDTLAKII-EI
HvDOG1L1     186 ------------------GGMDAAEGMEREMDAKAEAMKRVLEMADGLRLETLRGVV-GL
HvDOG1L2     156 -----------------------AGGELLDVGGLVGRVGAVVAGADALRLRTMKRAV-EI
HvDOG1L3     145 -----------------RQHPRNGVQADEAVAAVGQSLRVLLARGDALRERTVRGVV-RV
HvDOG1L5-2   401 -----------------SGNVASYMGQMAMAMGKLGTLENFLRQADNLRLQTLQQMQ-RI
HvDOG1L5-3   257 -----------------TGNVANYMGQMAMAMGKLGTLENFLRQADNLRQQTLQQMQ-RI
LesaDOG1     188 ---------------EHVGK--PNMVVDQALDKQEESMAKLLGEADNLRVETLVKIV-EI
LsDOG1       172 ---------------KGEGESSEGEVVDKAMDTHAPDLYNVLLEADKLRMKTLKGIL-EF
OsDOG1L1     180 --------------------------------------------DG--------------
OsDOG1L3     201 ------------------QHPRNG-EADEAVAAVGRSLRVLLAAADALRERTVRDVV-GT
OsDOG1L4     187 GA-SCDGLATRTRLVVATPEGAVDHEVDAAIGRYKAGLGRLLEEADELRMSTAQTLVTEI
OsDOG1L5-1   259 -----------------SGNVANYMGQMAMAMGKLGTLENFLRQADNLRQQTLHQMQ-RI
OsDOG1L5-3   389 -----------------ADNVTNYMGQMAIAMAKLTTLENFLRQADLLRHQTLQQMH-RI
OsDOG1L5-4   281 -----------------SSSPSNAADHTAAALGKIGAMESLLQQADDMRMQSLQKMQ-RV
SbDOG1L1     193 ---------------GGGGGGMDAGAMDREMQTKAEGMRQVLEMADGLRLETMREVV-AL
SbDOG1L2     163 -----------------------DGSGLLDVAGLVRTARAVLDRADALRLRTVKRAV-EI
SbDOG1L4     188 VRGSCGGAVRRLGVGAAGPGGGADAAVDAALDSYEAALARLLVEADELRMATSRTLATEI
SbDOG1L5     256 -----------------TGNVANYMGQMAMAMGKLGTLENFLRQADNLRLQTLQQMQ-RI
SoDOG1       191 ---------------EHVGE--PNVAVDQALDKQEEAMAALLAEADNLRVDTLSKII-EI
TaDOG1L1     186 ------------------GGVDAAEGMEREMDAKAEAMKRVLEMADALRLETLRGVV-GL
TaDOG1L2     156 -----------------------AGGELVDVGGLVGRVGAVLAGADALRLRTMKRAV-EI
TaDOG1L4     186 DR-SVKRDDIPGRLAAAESSGGLAAEVDAAMESYSAGLARLLEEADELRMSTARALATEI
TaDOG1L5-1   262 -----------------SGNVANYMGQMAMAMGKLGTLENFLRQADNLRQQTLHQMQ-RI
ZmDOG1L2     164 -----------------------DGSGGLDVAGLVRRARAVLDRADALRLRTVKRAV-EI
ZmDOG1L3     143 ------------------QPLRNG-EAEDAVAVVGRSLRVLLAAADALRDRTLRGVV-GL
ZmDOG1L4     188 ASGSCDGAARRR-LRAARLG-AADAEVDAALDSYKAALSRLLVEADELRMATARALATEI
ZmDOG1L5-1   255 -----------------TGNVANYMGQMAMAMGKLGTLENFLRQADNLRLQTLQQMQ-RI
ZmDOG1L5-3   331 --------------------PDPYMLQMATAVGILKELVNFVTQADHLRLTTLQQMH-KI
ZmDOG1L5-4   391 ---------------------MSFMSQMALALGKLANLEGFVIQADNLRQQTLHQMH-RI
```

```
AtDOG1        229 LSPVQGADFLLAGKKLHLSMHEWGTMRDRRRRDCMVDTEGNAGGEEGK------------
AtDOGL1       107 LTAVQAADFLLAGKKLHLAMHEWGKSREHRR---LEASGGDSGGNVTRE-----------
AtDOGL2       197 VTPLQAVEFLLAGKRLQLSLHDRGRVR---------ADVCGGVGGAAV------------
AtDOGL3       225 VTPVQAAEFLLAGKRLHISLHEWGRVREEQRFGCVRTDAAATGGAGTEKSKRSSLLM---
AtDOGL4       200 LTPPQCVEFLAAAATFQVQLRRWGNRRHYVTHS---------------------------
AtDOGL5       210 LSPLQQAEFLVAAAELRTGVAGWGTSHDRRRS---SEV----------------------
BdDOG1L1      223 LRPPQAVHFLLAAAELHLAVHHLGRRKDAHAHAPE-------------------------
BdDOG1L2      198 LEPAQAAELLVAMADMEIGFREFGLKHGDGGEPSRGA-----------------------
BdDOG1L3      219 LGPEQAGAFVAALLRFHLGVRRAGRGWSSGQGGQRGL-----------------------
BdDOG1L4      251 LTPRQAVEMLAAAKQLHLSICDWSRRKE-GAQSALLPPAATASSSSSGRPNA--------
BdDOG1L5-1    300 LTTRQSARALLAISGYFSRLRALSSLWIARPSTGMN------------------------
BdDOG1L5-3    368 LTTRQSARALLAISDYFSRLRALSSLWLARPRE---------------------------
BdDOG1L5-4    444 LTTRQSARALLAISDYFSRLRALSSLWLARPRE---------------------------
BrDOG1        233 LTPVQAGDFLMAGKKLHLSMHQWGALRDRRRRECIIDAGNDGGGEEEK------------
HvDOG1L1      227 LRPAQAVHFLVAAAELHLAVHKFGQHKDGAATAE--------------------------
HvDOG1L2      192 LEPAQASELLVAAADMEIGFREFGLKY-DGVG--AGGS----------------------
HvDOG1L3      187 VAPDPE------------------------------------------------------
HvDOG1L5-2    443 LTTRQSARALLAISDYFSRLRALSSLWLARPRE---------------------------
HvDOG1L5-3    299 LTTRQSARALLVISDYSSRLRALSSLWLARPKE---------------------------
LesaDOG1      230 LSPVEAANFLLAGKKLHLSMHEWGTMRDRRRRECIIEGGDKAKGEEEKKQ----------
LsDOG1        216 LTPLQAVEFLVAAKKLHLSLHEWSTRRDTRMGITQLLGGNNPSSSGDPPPET--------
OsDOG1L1          ------------------------------------------------------------
OsDOG1L3      241 LAPDQAGAFLAAMLRFHLGVHRAGRNWGSGNGGRRGL-----------------------
OsDOG1L4      246 LTPRQAVETLVAAKQLHLKVRSWSRRGE---AAVTQPARSPVTLPPAPSSNP--------
OsDOG1L5-1    301 LTIRQAARALLAIHDYFSRLRALSSLWLARPRE---------------------------
OsDOG1L5-3    431 LTTRQAARALLVISDYFSRLRALSSLWLARPRD---------------------------
OsDOG1L5-4    323 LTTRQSARALLLISDYFSRLRALNSLWIARPQQ---------------------------
SbDOG1L1      237 LRPAQAVHFLLAAAELHLAVHDFGRRKDGHAGNAAAAAAPPE------------------
SbDOG1L2      199 LEPAQAAELLVAAADLEIGFREFGLKH--GSG---RDG----------------------
SbDOG1L4      248 LTPRQAVEMLAAGKHLHLAVREWSRRREAGAAAAQQQLNGPSAGTADSSTSATVRRIGTH
SbDOG1L5      298 LTTRQSARALLVISDYSSRLRALSSLWLARPKE---------------------------
SoDOG1        233 LTPLQAADFLLAGKKLHLSMHEWGVLRDRRRRECIIDAVDDAGGKEEK------------
TaDOG1L1      227 LRPAQAVHFLVAAAELHLAVHKFGRRKDGAAAAE--------------------------
TaDOG1L2      192 LEPAQAAELLVAAADMEIGFREFGLKY-DGVG--AGGS----------------------
TaDOG1L4      245 LTPRQAVEMLMAAKQLHLAVRDWSRRKEEGAQNARLPRAAAATTAPSG-SNP--------
TaDOG1L5-1    304 LTIRQASRALLAIHDYFSRLRALSSLWLARPRE---------------------------
ZmDOG1L2      200 LEPAQAAELLVAAADLEIGFREFGLKH--GSG---RMT----------------------
ZmDOG1L3      183 LATDQAGAVVAAMLRFHLGVRRAGRDWSSGHGAQRRA-----------------------
ZmDOG1L4      246 LTPRQAVEMLAAGKHLHLSVRDWSRRREA-AAGAQQQLGGQSAGTDNTVRPST-------
ZmDOG1L5-1    297 LTTRQSARALLVISDYSSRLRALSSLWLARPKE---------------------------
ZmDOG1L5-3    370 LTPRQAARGLLALGDYFQRLRTLSSMWAARPREAAVS-----------------------
ZmDOG1L5-4    429 LTVRQAARCLLAIGEYHNRLRALSSLWASRPREILVTDEGNCEISIAAQPSQNQFSTF--
```
